# Recurrence Triangle Features for Unsupervised Time Series Clustering: Application to Human Gait

**DOI:** 10.3390/s26196137

**Published:** 2026-09-28

**Authors:** Md. Mehedi Hasan, Jun-ichiro Hirayama, Yoshiyuki Kobayashi, Masanori Shiro

**Affiliations:** 1 Degree Programs in Systems and Information Engineering, Graduate School of Science and Technology, University of Tsukuba, Tennodai, Tsukuba 305-8573, Ibaraki, Japan; 2 Human Informatics and Interaction Research Institute, National Institute of Advanced Industrial Science and Technology (AIST), Tsukuba 305-8568, Ibaraki, Japan; 3 Research Institute on Human and Societal Augmentation, National Institute of Advanced Industrial Science and Technology (AIST), Kashiwa 277-0882, Chiba, Japan; kobayashi-yoshiyuki@aist.go.jp

**Keywords:** recurrence plot, recurrence triangles, nonlinear dynamical systems, unsupervised learning, k-means clustering, time series clustering

## Abstract

**Highlights:**

**What are the main findings?**

- Recurrence triangle (RT) motifs capture fine-scale dynamical structure beyond global recurrence statistics.
- RT-based features improve unsupervised age-group discrimination from short gait time series.

**What are the implications of the main findings?**

- Local recurrence geometry yields interpretable representations of nonlinear dynamical structure.
- The proposed framework offers a simple yet effective approach to unsupervised analysis of short time series.

**Abstract:**

Unsupervised classification or clustering of complex time series generated from nonlinear dynamical systems remains challenging, particularly when signals exhibit noise and subtle structural differences. We develop a recurrence-based framework that captures local geometric patterns in recurrence plots by extracting triangular motifs, termed recurrence triangles (RTs), and mapping their relative frequencies to compact feature vectors for clustering. The approach is evaluated on four synthetic systems: the continuous-time Rössler and Lorenz systems and the discrete-time Logistic map and AR(2) model. RT-based features generally achieved higher clustering accuracy than classical recurrence quantification analysis (RQA), although performance depended on the dynamical system and signal length. RQA performed better than RT for AR(2) at longer signal lengths, while a statistical baseline, namely StatACF, also outperformed RT under several conditions, indicating that no single representation was uniformly optimal. We further applied the methods to gait recordings from younger and older adults. RT features achieved the highest observed clustering accuracy among the three representations (73.7%), followed by StatACF (68.4%) and RQA (52.6%). However, the differences among the methods were not statistically significant in the small cohort (*n* = 19). RT motif distributions also differed between age groups in a marker-dependent manner. These findings suggest that RT distributions capture fine-scale recurrence structure complementary to global recurrence statistics and warrant further validation in larger, independent cohorts.

## 1. Introduction

Analyzing time-series data generated by nonlinear dynamical systems is central to a wide range of scientific and engineering domains, including biomedical signal processing, climate modeling, and financial forecasting [1,2,3]. In many practical scenarios, the task is to determine whether observed time series originate from distinct underlying dynamical regimes. This problem becomes particularly challenging when data are short, noisy, and unlabeled. Sensitivity to initial conditions and parameter variations further complicates the use of conventional linear or statistical descriptors, while machine learning methods rely critically on feature representations that preserve meaningful dynamical structure.

A broad spectrum of time-series analysis techniques has been developed to address these challenges. Symbolic approaches, such as ordinal pattern analysis and permutation entropy, discretize temporal structure into symbolic sequences, providing robustness to noise and computational efficiency [4,5,6,7]. Transformation-based methods, including Fourier, wavelet, and multiscale representations, characterize signals through spectral or scale-dependent summaries and have seen widespread application [8,9,10]. More recently, deep learning and related representation learning methods have achieved remarkable success in time-series classification by automatically extracting features from raw data [11]. Despite their strong empirical performance, such approaches typically require large labeled datasets, extensive training, and careful hyperparameter tuning, and they offer limited interpretability. These requirements limit their suitability for scientific settings where data are usually scarce, and understanding how and why dynamics differ is as important as classification accuracy.

In this context, methods grounded in nonlinear dynamical systems theory offer a complementary perspective. Rather than relying on basic statistical summaries or learned representations, these approaches aim to characterize time series through the geometry of their trajectories in state space, thereby directly reflecting properties of the underlying dynamics. Such geometric descriptions are inherently model-free, require minimal training, and remain meaningful even for short or noisy observations. By focusing on recurrence—the tendency of a system to revisit similar states over time—these methods provide a natural bridge between dynamical systems theory and data-driven analysis [12]. Recurrence-based approaches have also been applied to physiological signals such as human gait, where they have demonstrated the ability to distinguish pathological gait patterns from healthy controls [13]. This focus motivates the present study’s emphasis on recurrence-based representations as a principled and interpretable foundation for unsupervised time-series discrimination.

Recurrence plots (RPs), introduced by Eckmann et al. [14], provide a powerful visualization of when a dynamical system revisits similar states in phase space. Since their introduction, RPs have been widely used for analyzing nonlinear dynamics in physical, biological, and physiological systems [12,15,16,17,18]. Building on this idea, recurrence quantification analysis (RQA) summarizes RPs into global metrics such as determinism, laminarity, average diagonal line length, and maximum diagonal line length [12,15]. These features have not only been used for descriptive analysis but also as inputs to classification and clustering frameworks. In particular, RQA-based features have been successfully employed in supervised classification tasks involving biomedical signals, including EEG-based detection of epileptic activity and gait-based discrimination of neurological disorders, where they are combined with machine learning classifiers to distinguish between dynamical regimes [19,20,21,22]. However, because RQA aggregates recurrence information into global statistical measures, it inherently loses localized geometric structures embedded within recurrence patterns. Several studies have noted that such global descriptors may be insufficient to capture subtle dynamical differences, especially in short, noisy, or high-dimensional time series, thereby limiting their discriminative capability in more challenging classification or unsupervised clustering scenarios [23,24,25].

To address this limitation, we propose a novel framework based on the recurrence triangle (RT) that explicitly encodes local recurrence geometry within RPs. Building on the formulation introduced by Hirata [26], we enumerate triangular motifs formed by neighboring recurrence points and summarize their central tendency while discarding low-probability triangular motifs. This construction yields compact, interpretable feature vectors, in which each component provides a probability-weighted compressed representation of the dominant local recurrence configurations. By design, the RT framework captures micro-scale dynamical organization and provides a compact representation that can be evaluated on relatively short time series.

We validate the proposed approach on four representative synthetic systems—continuous-time Rössler and Lorenz systems, and discrete-time Logistic map and AR(2) process—by comparing RT-based features with classical RQA descriptors and a baseline based on statistical and autocorrelation features (StatACF). RT-based features generally provided stronger discrimination than RQA, although this advantage was system-dependent, while StatACF outperformed RT under several conditions. We further demonstrate the applicability of the approach to real-world human gait dynamics. RT features achieved the highest observed clustering accuracy among the three representations tested, although this difference did not reach statistical significance in this small cohort.

The main contributions of this study are

- A localized recurrence-geometry framework that captures fine-scale dynamical structure beyond global RQA statistics.
- An interpretable RT-based feature mapping that supports unsupervised learning on short time series.
- Comprehensive evaluation on synthetic and physiological data, including comparisons with classical recurrence-based descriptors and statistical/autocorrelation-based descriptors.

The remainder of this paper is organized as follows. Section 2 presents background and methodology, Section 3 details experimental results, Section 4 discusses the results, and Section 5 concludes.

## 2. Methods

This section outlines the proposed methodology for time series clustering using recurrence triangles (RTs) derived from RPs. We begin with the construction of RPs, followed by the definition and extraction of RTs to capture local recurrence patterns. Subsequent steps include computing the relative frequencies of RT motifs, robustly transforming these into feature vectors and applying k-means clustering. The methodology is detailed through mathematical formulations and a step-by-step procedure, complemented by illustrative figures (Figure 1, Figure 2 and Figure 3) to clarify the process.

### 2.1. Recurrence Plot Construction

Given a *d-*dimensional time series ${\left\{x_{t}\right\}}_{t=1}^{N}$, where $x_{t}\;\epsilon R^{d}$, the recurrence plot (RP) is defined as

$$R_{i,j}=\Theta \left(\varepsilon -∥x_{i}-x_{j}∥\right),i,j=1,2,\ldots ,N$$

where $\Theta \left(.\right)$ is the Heaviside step function, ε is a recurrence threshold, and ∥.∥ is the Euclidean norm [12]. Figure 1 illustrates representative examples of RPs. The RP encodes pairwise recurrences as a binary matrix. The recurrence rate (RR), defined as the density of recurrence points in the RP, is given by $RR=\;\frac{1}{N(N-1)}\;\sum_{i\neq j}^{N}R_{i,j}$. In this study, the threshold ε is selected such that *RR* = 10%, excluding the line of identity, following common practice to balance sparsity and structural visibility in RPs across different datasets [27]. RPs provide not only a visualization of state recurrences but also qualitative insight into the underlying dynamics of a system. The geometric structures appearing in an RP are closely related to dynamical properties. These dynamical properties are briefly described in Section 2.5.

In the present analysis, the RPs are constructed directly from the observed state vectors without delay-coordinate or phase-space reconstruction. Consequently, no embedding dimension or time delay is introduced. No Theiler window is applied; recurrence is evaluated directly between pairs of observed state vectors. The Euclidean distance is used to quantify the distance between state vectors.

### 2.2. Recurrence Triangles (RTs)

Recurrence triangles (RTs) are localized geometric structures identified within RPs, originally proposed by Hirata [26]. An RT corresponds to a small triangular region of the RP, as illustrated in Figure 2, which encodes short-term recurrence behavior that global RP measures may not capture. RTs are particularly useful because they represent the smallest structure involving three time points, thereby extending beyond pairwise recurrences. This enables RTs to capture higher-order dynamical patterns that are not reflected in individual recurrence points or global RP-based statistics.

Formally, an RT of size *L* at position (*i*, *j*) in an RP is defined as $RT\left(i,\;j,\;L\right)=\;\left\{R_{a,\;b}|\;a=i,\;i+1,\;\ldots ,\;i+L-2;b=j+\left(a-i\right)+1,\;j+\left(a-i\right)+2,\;\ldots ,\;j+L-1\right\}$, where L ≥2, and 1 ≤i, j ≤N−L+1.

The size *L* of an RT corresponds to the number of consecutive time indices considered in its construction. For a given size *L*, all pairwise recurrence relations $R\left(a,\;b\right)$ with a<b are included, resulting in a total of $E=\;L\;\left(L-1\right)/2$ binary elements. Each element represents a pairwise recurrence relation between two-time indices and takes a value of either 1 (recurrence) or 0 (non-recurrence). Consequently, the total number of possible RT motifs is $T=\;2^{E}$. Thus, each RT corresponds to one of *T* distinct binary configurations.

### 2.3. RT Motifs as Feature Vectors

Given a time series of interest, we extract all RTs of size *L* from its RP and count the frequency of each configuration. Then, normalizing the frequency by the total number of RTs yields the probability vector

$$P=\left[p_{1},p_{2},\ldots ,p_{T}\right],\sum_{k=1}^{T}p_{k}=1.$$

Each motif k is represented by a binary vertex vector $u_{k}=(u_{k1},\;u_{k2},\;\ldots ,\;u_{kE})$, with $u_{kj}\in \left\{0,\;1\right\}$, where $u_{kj}=1$ indicates the presence of the *j-*th recurrence relation.

To map the motif probability distribution to a feature vector, we use a probability-weighted accumulation of the motif vertex vectors. In the full representation, the *j*-th feature is

$$f_{j}=\sum_{k=1}^{T}p_{k}u_{kj},\;j=1,2,\;\ldots ,\;E$$

To restrict the representation to the dominant RT motifs, we introduce a top-*M* accumulation rule. Let $p_{\left(1\right)}\geq p_{\left(2\right)}\geq \ldots \geq p_{\left(T\right)}$ denote the motif probabilities sorted in descending order, with corresponding vertex vectors $u_{\left(1\right)}$, …, $u_{\left(T\right)}$. Let $I_{M}$ denote the indices of the *M* motifs with the largest empirical probabilities. The resulting feature vector is defined as

$$f_{j}=\sum_{k\in I_{M}}p_{k}u_{kj},\;j=1,2,\;\ldots ,\;E$$

Thus, the feature vector does not consist of the probabilities of the top-*M* motifs themselves. Instead, the probabilities of the selected motifs are accumulated according to the recurrence relations represented by their vertex vectors. The parameter *M* therefore controls the number of dominant motifs contributing to the feature representation.

The value of *M* is determined through a sensitivity analysis. Clustering performance is evaluated over a range of candidate *M* values using repeated independent trials. The candidate with the highest mean clustering accuracy is selected. The consistency of this selection is then checked using independent subsets of the repeated trials. The sensitivity analysis, the resulting per-system values of *M*, and this stability check are reported in the section titled Selecting the top-*M* Parameter. In this study, *L* = *3* is used as the default RT size. For an RT of size *L*, the number of pairwise recurrence relations is E=L(L−1)/2. This gives $2^{E}$ possible RT motifs. Increasing *L* increases both the feature dimensionality *E* and the size of the motif space *T*. This allows richer local structure to be captured. However, for a fixed signal length, the same number of observed RT instances is spread across more possible configurations. This produces sparser motif counts. Probability estimates may therefore become less reliable. Larger *L* can capture more detailed structure. Smaller *L* provides a more compact representation with denser motif statistics. Feature construction also becomes more computationally demanding as *L* increases. Runtime was not directly measured in this study, so the computational trade-off associated with increasing *L* is discussed qualitatively rather than evaluated empirically.

### 2.4. Unsupervised Clustering by K-Means

After constructing feature vectors from all the time series given in a dataset, we perform unsupervised clustering using the k-means algorithm [28]. Before clustering, each entry of the feature vector is standardized to have mean zero and unit variance (z-score normalization). The algorithm partitions the set of standardized feature vectors into *k* subsets (clusters) by minimizing the within-cluster sum of squares:

$$\underset{C_{1},C_{2},\ldots ,C_{k}}{\min}\sum_{i=1}^{k}\sum_{V\in C_{i}}{∥V-\mu_{i}∥}^{2},$$

where $C_{i}$ denotes the *i*-th cluster and $\mu_{i}=\sum_{V\in C_{i}}V/\left|C_{i}\right|$ its centroid.

For evaluation, we compare the obtained cluster indices with given class labels (unused for clustering) as the ground truth. Since the correspondence between cluster indices and class labels is unknown in advance, we select the label assignment that maximizes the classification accuracy and then report the maximum accuracy as the measure of clustering performance. The summary of the overall procedure is given in Figure 3.

### 2.5. Baseline Method: Classical RQA Features

To benchmark the proposed framework, we use a classical RQA feature set as a baseline [12]. RQA summarizes RP structures into interpretable scalar measures, providing insight into system dynamics and enabling feature-based classification.

Let $R\in {\left\{0,\;1\right\}}^{N\times N}$ denote the RP of a time series of length *N*, and let *P*(*l*) and *P*(*v*) represent the histograms of diagonal and vertical line lengths, respectively, with minimum line lengths $l_{min}$ and $v_{min}$. Then, typical RQA features are given as follows:

1. Determinism (DET) measures the proportion of recurrence points that align along diagonal lines in an RP, reflecting the predictability of the system. Mathematically, it can be written as

$$DET=\frac{\sum_{{l=l}_{min}}^{N}lp\left(l\right)}{\sum_{i,\;j=1}^{N}R(i,\;j)}$$

High DET indicates predictable dynamics, while low DET suggests irregular or chaotic behavior.

2. Average diagonal line length (L) quantifies the mean length of diagonal structures in the RP, capturing the typical duration of deterministic episodes. Mathematically,

$$L=\frac{\sum_{{l=l}_{min}}^{N}lp\left(l\right)}{\sum_{{l=l}_{min}}^{N}p\left(l\right)}$$

Longer average diagonal lines indicate sustained regularity.

3. Longest diagonal line ($L_{max}$) represents the maximum length of diagonal lines, which is indicative of the longest uninterrupted deterministic sequence in the system. Mathematically,

$$L_{max}=max\left\{l\;|\;p\left(l\right)>0\right\}$$

$L_{max}$ captures system stability and sensitivity to initial conditions.

4. Laminarity (LAM) measures the fraction of recurrence points forming vertical lines, characterizing the presence of laminar phases where the system remains in similar states. Mathematically,

$$LAM=\frac{\sum_{{v=v}_{min}}^{N}vp\left(v\right)}{\sum_{i,j=1}^{N}R\left(i,j\right)}$$

Higher *LAM* values reflect extended laminar phases in the system.

5. Entropy characterizes the diversity of diagonal line lengths and quantifies the complexity of deterministic structures. Mathematically,

$$ENTR=-\sum_{{l=l}_{min}}^{N}P\left(l\right)logP(l)$$

where $P\left(l\right)=\;p(l)/\sum_{{l=l}_{min}}^{N}P\left(l\right)$

6. Trapping time (TT) represents the average length of vertical line structures and characterizes the typical duration of laminar states:

$$TT=\frac{\sum_{{v=v}_{min}}^{N}vp\left(v\right)}{\sum_{{v=v}_{min}}^{N}p\left(v\right)}$$

To perform unsupervised clustering, we apply k-means to the standardized feature vectors and evaluate its performance in the same manner as above. All six RQA features are combined into a feature vector for each time series: $f=\;\left[DET,\;L,\;L_{max},\;LAM,\;ENTR,\;TT\right]$. This approach provides a baseline for comparison against the RT-based method.

### 2.6. Baseline Method: Statistical and Autocorrelation-Based Features

To provide a baseline independent of recurrence-plot representations, we constructed a feature set from conventional statistical and autocorrelation-based descriptors denoted StatACF. This follows the characteristic-based approach of representing time series using global statistical descriptors for clustering [9]. Unlike RQA and RT, these features are calculated directly from the original time series. The baseline was therefore used to assess whether RT features provide information beyond general statistical characteristics and short-range temporal dependence.

Let $x={\left\{x_{t}\right\}}_{t=1}^{N}$ denote a scalar time series of length *N*, with sample mean $\mu =\;\frac{1}{N}\;\sum_{t=1}^{N}x_{t}$. Four statistical descriptors were calculated: mean, standard deviation, skewness, and kurtosis. The standard deviation was calculated as

$$\sigma =\sqrt{\frac{1}{N-1}\sum_{t=1}^{N}{\left(x_{t}-\mu \right)}^{2}}$$

Skewness and kurtosis were calculated as the standardized third and fourth central moments, respectively:

$$\gamma_{1}=\;\frac{\frac{1}{N}\;\sum_{t=1}^{N}{\left(x_{t}-\mu \right)}^{3}}{{\left[\frac{1}{N}\;\sum_{t=1}^{N}{\left(x_{t}-\mu \right)}^{2}\right]}^{3/2}},$$

and

$$\gamma_{2}=\frac{\frac{1}{N}\;\sum_{t=1}^{N}{\left(x_{t}-\mu \right)}^{4}}{{\left[\frac{1}{N}\;\sum_{t=1}^{N}{\left(x_{t}-\mu \right)}^{2}\right]}^{2}}$$

Here, the kurtosis is defined such that a Gaussian distribution has a kurtosis of 3.

To characterize short-range temporal dependence, the first ten autocorrelation coefficients were additionally calculated. For lag k, the autocorrelation was defined as

$$\rho_{k}=\frac{\sum_{t=1}^{N-k}\left(x_{t}-\mu \right)\left(x_{t+k}-\mu \right)}{\sum_{t=1}^{N}{\left(x_{t}-\mu \right)}^{2}},\;k=1,\;2,\;\ldots ,\;10.$$

The resulting feature vector for each scalar time series was therefore

$$f_{StatACF}=\left[\mu ,\;\sigma ,\;\gamma_{1},\;\gamma_{2},\rho_{1},\;\ldots ,\;\rho_{10}\;\right]\;\epsilon R^{14}.$$

## 3. Results

### 3.1. Results on Toy Models

In this section, we evaluate the proposed RT-based feature extraction and unsupervised clustering framework on four representative toy models, including both continuous-time and discrete-time dynamical systems. We first describe the generation of time series data for the Rössler and Lorenz continuous systems, followed by the Logistic map and second-order autoregressive (AR(2)) processes’ discrete-time models. For each system, we explain the parameters used, the feature construction procedure, and the application of the top-*M* accumulation rule. Finally, we present the clustering performance obtained using *k*-means and visualize the results with scatter plots, highlighting the accuracy and separability of the generated features. Table 1, Table 2 and Table 3 summarize the clustering accuracy across all toy models.

#### 3.1.1. Data Generation

The first model considered is the Rössler system [29], defined as

(1) $$\frac{dx}{dt}=-y-z+{\gamma \eta }_{t}$$

(2) $$\frac{dy}{dt}=x+ay+\;\gamma \eta_{t}\;$$

(3) $$\frac{dz}{dt}=b+z\;\left(x-c\right)+{\gamma \eta }_{t}$$

where $\eta_{t}$ is a Gaussian random variable with mean 0 and unit variance, independently sampled for each *t*. Following the dynamical parameter selection strategy of [25], parameters are chosen to ensure operation within well-characterized chaotic regimes while avoiding transitions to periodic behavior. The parameters a = 0.2, c = 5.7 are fixed, and variability is introduced by varying the control parameter b. Two datasets are generated by sampling b from non-overlapping chaotic intervals identified via bifurcation analysis. Specifically, for Dataset 1, we use $b\in \;\left[0.37,\;0.44\right]$, while for Dataset 2, we use $b\in \;\left[0.46,\;0.49\right]$. These intervals correspond to dynamically distinct chaotic regimes, as demonstrated in [25]. Additional dynamical analyses, including Lyapunov exponent estimation and spectrum convergence, are provided in Appendix A to further verify that the selected parameter ranges correspond to distinct dynamical regimes. Stochastic perturbations are incorporated directly into the dynamical equations at a fixed noise strength of γ=0.1. For each dataset, 30 parameter values are sampled uniformly within the specified intervals. For each parameter setting, 50 independent trials are simulated using randomized initial conditions $\left[0.1,\;0.1,\;0.1\right]+10^{-2}.N(0,\;1)$, to evaluate statistical variability and classification robustness. Time series of lengths 200, 400, …, 1000 are generated for each realization.

The second continuous-time system is the Lorenz system [30], given by

(4) $$\frac{dx}{dt}=\sigma \left(y-x\right)+\gamma \eta_{t}$$

(5) $$\frac{dy}{dt}=x\left(\rho -z\right)-y+{\gamma \eta }_{t}\;$$

(6) $$\frac{dz}{dt}=xy-\beta z+{\gamma \eta }_{t}$$

where $\eta_{t}$ is a Gaussian random variable with mean 0 and unit variance and γ represents the noise intensity. In this study, the noise intensity is fixed at γ=0.1. The parameters σ=10, $\beta =\;\frac{8}{3}$ are fixed, corresponding to the canonical Lorenz configuration originally introduced by Lorenz [30]. The control parameter ρ governs the qualitative behavior of the system and induces a rich bifurcation structure, including transitions between steady, periodic, and chaotic dynamics [31]. Based on this bifurcation structure, two non-overlapping parameter intervals within the chaotic regime are selected to construct datasets exhibiting distinct dynamical characteristics. Specifically, for Dataset 1, we use $\rho \in \;\left[25,\;27\right]$, while for Dataset 2, we use $\rho \in \;\left[28,\;30\right]$, both sampled at equal intervals. Although both ranges correspond to chaotic dynamics, they occupy different regions of the bifurcation diagram and are associated with distinct attractor geometries and temporal recurrence structures. To ensure that the selected parameter ranges remain within the chaotic regime and do not intersect periodic windows, additional analyses—including bifurcation diagrams, Lyapunov exponent estimation, and sensitivity to initial conditions—are performed and reported in Appendix B. For each dataset, 30 parameter values are sampled uniformly within the specified intervals. For each parameter setting, 50 independent trials are simulated using randomized initial conditions $\left[0.1,\;0.1,\;0.1\right]+10^{-2}.N(0,\;1)$, to evaluate statistical variability and classification robustness. Time series of lengths 200, 400, …, 1000 are generated for each realization.

For both continuous models, we numerically solved the differential equations using the ode45 solver in MATLAB (version R2026a; The MathWorks, Inc., Natick, MA, USA), an adaptive-step embedded Runge–Kutta method (Dormand-Prince). The Gaussian perturbations were generated within the derivative function evaluated by the solver. Consequently, the perturbations were resampled at the solver’s internal function evaluations rather than being generated once at prescribed output sampling times. This implementation was used consistently across all parameter values and independent trials. It is therefore treated as a heuristic stochastic perturbation scheme rather than as a formal discretization of a stochastic differential equation. The resulting trajectories were sampled at a uniform interval of ∆t=1. The first 1000 sampled points of each trajectory were discarded to remove transient effects.

The third model is the Logistic map [32], defined as follows

(7) $$x_{t+1}=rx_{t}\left(1-x_{t}\right)$$

The control parameter *r* governs the qualitative behavior of the map, inducing transitions from periodic to chaotic dynamics through a well-known cascade of bifurcations. We selected two non-overlapping parameter intervals to construct two datasets with different parameter ranges [33,34]. Specifically, Dataset 1 uses $r\in \;\left[3.56,\;3.60\right]$, while Dataset 2 uses $r\in \;\left[3.67,\;3.70\right]$, with parameter values sampled at equal intervals. The dynamical regimes within these parameter ranges were subsequently examined using direct numerical Lyapunov-exponent calculations, as described in Appendix C.

The fourth model is the following second-order autoregressive (AR(2)) model [35]. The model is defined as

(8) $$x_{t+1}=a_{1}x_{t}+a_{2}x_{t-1}+{\sigma_{noise}\eta }_{t}\;$$

where $a_{1}$ and $a_{2}$ are autoregressive coefficients, $\eta_{t}$ denotes Gaussian noise, and $\sigma_{noise}=0.1$ is the noise standard deviation. The qualitative temporal behavior of an AR(2) process is determined by the roots of its characteristic polynomial $1-\;a_{1}z-\;a_{2}z^{2}=0$. Specifically, when the discriminant $∆\;=\;a_{1}^{2}+4a_{2}\geq 0$, the roots are real, and the autocorrelation function exhibits a monotonic, non-oscillatory exponential decay. In contrast, when ∆ <0, the roots form a complex-conjugate pair, leading to damped oscillatory dynamics with a characteristic frequency [35]. Based on this criterion, two distinct parameter regimes were selected. In the first regime, $a_{1}\in \;\left[0.3,\;0.45\right]$ and $a_{2}\in \;\left[0.02,\;0.035\right]$, ensuring ∆ >0 and producing non-oscillatory, purely damped dynamics. In the second regime, $a_{1}\in \;\left[0.8,\;0.95\right]$ and $a_{2}\in \;\left[-0.35,\;-0.25\right]$, for which ∆ < 0, resulting in stable damped oscillations. For each dataset, 30 parameter values are sampled uniformly within the specified intervals. For each parameter setting, 50 independent trials are simulated using randomized initial conditions $0.1\;+10^{-2}.N(0,\;1)$, to evaluate statistical variability and classification robustness. Time series of lengths 200, 400, …, 1000 are generated for each realization.

For both discrete models, the time series were generated by direct iteration of their respective difference equations. The first 1000 points of each generated sequence were discarded to remove transient effects.

#### 3.1.2. Baseline: Results Using RQA Features

To establish a baseline, we first evaluated the discriminative capability of classical recurrence quantification analysis (RQA) features. Six standard measures were considered: $\left(a\right)$ determinism (DET), (b) laminarity (LAM), (c) average diagonal line length (L), (d) maximum diagonal line length ($L_{max}$), (e) entropy (ENTR), and (f) trapping time (TT). For each time series across all toy models, RPs were constructed at a fixed recurrence rate of 10%, and RQA features were computed using standard minimal line-length thresholds. The resulting six-dimensional feature vectors were z-standardized and subjected to *k*-means clustering (*k* = 2). Since clustering labels are arbitrary, cluster assignments were optimally matched to the ground-truth classes for evaluation.

Table 1 reports the clustering accuracy obtained using RQA features. A formal statistical comparison between RQA, RT, and StatACF is presented in Section 3.1.6.

For the Rössler system (Table 1), clustering accuracy remains consistently above chance level, with mean values ranging from 56.0% to 65.0%. Performance improves modestly with increasing length, reaching its maximum at 1000 samples. However, the relatively large standard deviations (≈5.6–8.1%) indicate considerable variability across trials, suggesting limited robustness of global RQA descriptors for distinguishing nearby chaotic regimes. The Lorenz system (Table 1) exhibits a flatter accuracy profile. Mean accuracies remain confined to a narrow range between 54.1% and 56.2% across all lengths, with comparatively smaller variability. This indicates that increasing the observation window provides little additional discriminative information when using classical RQA features for the selected Lorenz parameter settings. For the Logistic map (Table 1), the highest mean accuracy (61.8%) is observed at the shortest length (200 samples). As the length increases, accuracy decreases and stabilizes near 55–56%, while the standard deviation drops sharply. This behavior suggests that short trajectories may transiently reflect parameter-specific fluctuations, whereas longer realizations converge toward statistically similar recurrence structures characteristic of fully developed chaos. In contrast, the AR(2) process (Table 1) demonstrates a strong monotonic increase in clustering accuracy, rising from 81.4% at 200 samples to 98.2% at 1000 samples, accompanied by steadily decreasing variability. This reflects the fundamentally different nature of linear stochastic dynamics, where global correlation structure dominates and is effectively captured by line-based RQA statistics.

Overall, these results indicate that classical RQA features are effective for systems dominated by strong global temporal structure (e.g., AR processes), but show limited sensitivity when class differences manifest as localized or subtle geometric distortions in chaotic attractors. This limitation motivates the use of more localized recurrence descriptors.

#### 3.1.3. Baseline: Results Using StatACF Features

As a second baseline, we evaluated the performance of the StatACF feature set described in Section 2.6. For each time series across the four toy models, the features were calculated directly from the signal, standardized using z-score normalization, and subjected to *k*-means clustering with *k* = 2, following the procedure described in Section 2.4. The resulting clustering accuracies are summarized in Table 2.

For the Rössler system, the clustering accuracy increased substantially with data length, from 59.9 ± 5.6% at 200 samples to above 93% for lengths of 400 samples and longer, reaching 95.4 ± 3.1% at 1000 samples. The variability across trials also decreased at longer data lengths.

The Lorenz system showed a non-monotonic dependence on data length. Accuracy varied between 55.7% and 83.7%, with relatively high performance at 400 and 800 samples but substantially lower accuracy at 600 and 1000 samples. This indicates that increasing the data length did not consistently improve the performance of the StatACF representation for this system.

For the Logistic map, StatACF achieved consistently high clustering accuracy across all tested data lengths, remaining above 98% throughout the experiment. The small standard deviations indicate stable performance across repeated trials.

For the AR(2) process, accuracy generally increased with data length, reaching 83.0 ± 14.7% at 800 samples before slightly decreasing at 1000 samples. Compared with the other systems, the substantially larger standard deviations indicate greater variability in classification accuracy across repeated trials.

Overall, the StatACF baseline provided strong separation for the Logistic map and, at longer data lengths, for the Rössler and AR(2) systems, whereas its performance was less consistent for the Lorenz system. These results provide a non-recurrence baseline for evaluating the performance of the RT-based representation.

#### 3.1.4. Sensitivity Analysis

We tested three parameters for robustness: the top-*M* parameter, the triangle size *L*, and the recurrence rate *RR*. Each analysis varied one parameter while holding the others at their default values (*L* = 3, *RR* = 10%, and each system’s selected *M*). All analyses used 50 independent trials, 30 datasets per class, and time series of length 600.

##### Selecting the Top-*M* Parameter

The number of retained top motifs *M* was varied from 1 to 8 for each system (Figure 4). At *M* = 1, the clustering accuracy was close to the chance level expected under random cluster-label assignment for all four systems. For M>1, the clustering accuracy varied substantially across the tested values and reached a maximum at a system-specific value of *M*.

The Rössler system and the Logistic map achieved their highest clustering accuracies at *M* = 3, whereas the Lorenz system and the AR(2) process achieved their highest accuracies at *M* = 4. For the Lorenz system, the accuracy at *M* = 3 was close to the level expected under random cluster-label assignment, in contrast to the higher accuracy obtained at *M* = 4. The selected value of *M* provided significantly higher clustering accuracy than each of the other tested values after Holm correction for multiple comparisons ($p_{Holm}<0.001$ for all comparisons; paired Cohen’s d ranged from 0.76 to 1.63). These results indicate that the value of *M* yielding the highest clustering accuracy differs among the dynamical systems. The selection was further examined using a split-half stability analysis. The 50 trials were divided into two non-overlapping groups of 25 trials, and the value of *M* yielding the highest mean accuracy was determined separately for each group. The same value of *M* was selected in both groups for all four systems: *M = 3* for the Rössler system and the Logistic map and *M = 4* for the Lorenz system and AR(2) process. This agreement provides additional evidence that the selected *M* values were not sensitive to the particular subset of trials used. We note that *M* was selected using ground-truth class labels, since candidate values were compared by their resulting clustering accuracy. The clustering procedure itself remains unsupervised. However, the choice of *M* is label-informed rather than fully label-free. This sensitivity analysis was performed at a signal length of 600. The selected M was then applied, without further adjustment, to independently generated trials at the other tested lengths (200, 400, 800, and 1000 samples). The reported performance at those lengths is therefore not affected by this dependency. At length 600 specifically, the same trials were used both to select *M* and to report performance at that length.

##### Sensitivity to Triangle Size

The sensitivity to triangle size *L* was evaluated using the system-specific values of *M* selected in the section titled Selecting the top-*M* Parameter, while the recurrence rate was fixed at *RR* = 0.10. The triangle size was varied from *L* = 2 to *L* = 5 (Figure 5). An RT of size *L* contains E=L(L−1)/2 recurrence edges and therefore $2^{E}$ possible binary motif configurations. Thus, increasing L substantially increases the size of the motif space.

The effect of *L* differed among the four systems. The Logistic map and AR(2) process achieved their highest clustering accuracy at *L* = 3, followed by a marked decrease at larger triangle sizes. In contrast, the Rössler system showed a progressive increase in accuracy across the tested range, with the highest accuracy at *L* = 5. The Lorenz system showed a similar overall increase, although a small decrease was observed from *L* = 3 to *L* = 4, before accuracy increased again at *L* = 5. Thus, larger triangle sizes improved clustering for the Rössler and Lorenz systems within the tested range, whereas *L* = 3 provided the highest accuracy for the Logistic system and AR(2) process.

These results indicate that the effect of triangle size is system-dependent. Therefore, *L* = 3 was not selected because it universally maximized clustering accuracy. Instead, it was retained for the main analysis because it provides a compact three-edge representation with only eight possible motifs, whereas *L* = 4 and *L* = 5 produce 64 and 1024 possible motifs, respectively. This choice provides a consistent and relatively compact representation across the four systems.

##### Sensitivity to Recurrence Rate

The sensitivity to the recurrence rate was evaluated using *L* = 3 and the system-specific values of *M* selected in the section titled Selecting the top-*M* Parameter. The recurrence rate was varied from *RR* = 0.05 to 0.40 (Figure 6). For each value of *RR*, the recurrence threshold was determined separately for each time series to achieve the prescribed recurrence rate.

The classification accuracy increased with *RR* over part of the tested range for all four systems, but the response differed among the systems. The Rössler system showed its highest accuracy at *RR* = 0.20, while the Lorenz system continued to improve up to RR = 0.30. The Logistic map showed a strong increase in accuracy from *RR* = 0.05 to higher recurrence rates and reached 100% at *RR* = 0.40. In contrast, the AR(2) process reached its highest accuracy at *RR* = 0.20, followed by a marked decrease at *RR* = 0.30 and *RR* = 0.40.

These results indicate that classification performance depends on the recurrence rate and that the highest-performing value differs among the dynamical systems. Therefore, *RR* = 0.10 should not be interpreted as a universally optimal setting. Instead, *RR* = 0.10 was retained for the main analysis as a common reference value, allowing the RT representation to be evaluated under the same recurrence-rate setting across all systems.

#### 3.1.5. Results Using the Proposed Method for Synthetic Systems

For each time series, RPs were constructed using a fixed recurrence rate of 10%, ensuring comparable recurrence densities across all datasets. Recurrence triangles (RTs) were then extracted to characterize localized geometric structures within the RPs.

For all dynamical systems considered—including the Rössler system, Lorenz system, Logistic map, and AR(2) model—we employed a uniform triangle size of *L* = 3. This choice provides 8 possible RT configurations, which provide a compact and expressive representation of local recurrence geometry. For each time series, the probability distribution over the eight RT motifs was computed. The *M* most probable RT motifs were selected—with *M* determined per system via the sensitivity analysis in the section titled Selecting the top-*M* Parameter—and their probability-weighted contributions were accumulated according to the formulation in Section 2.3 to form the feature vector $f^{\left(i\right)}=[f_{1},\;f_{2},\;f_{3}]$, for the *i*-th time series, where each component $f_{j}$ corresponds to one of the *E* = 3 pairwise recurrence relations between the three consecutive time indices defining the *L* = 3 triangle. Thus, the three components of $f^{\left(i\right)}$ are not the probabilities of the selected top-*M* motifs themselves; rather, they are the probability-weighted accumulated contributions of those motifs over the three recurrence relations. The resulting feature vectors were standardized using z-score normalization and clustered using *k*-means with *k* = 2. Since the cluster labels are arbitrary, assignments were aligned with ground-truth classes to evaluate clustering accuracy. By employing a consistent triangle size and feature dimensionality across all models, this experimental design enables a fair and direct comparison of discriminability across continuous-time and discrete-time systems, while isolating the contribution of localized recurrence geometry captured by the RT framework.

The clustering accuracy obtained using RT-based features is summarized in Figure 7 and Table 3.

For the Rössler system (Figure 7a), accuracy increases from 72.7% at 200 samples to a peak of 84.7% at 600 samples, followed by a slight decline for longer sequences while remaining above 80%. Variability is high at short lengths but stabilizes near 5% as length increases, indicating improved robustness once sufficient recurrence structure is captured. The Lorenz system (Figure 7b) exhibits pronounced non-monotonic behavior. Near-perfect accuracy is achieved at 200 samples (99.1%), followed by a sharp drop at 400 samples (58.0%), and subsequent recovery to near-perfect performance at 800 and 1000 samples. Standard deviations remain low across all lengths, indicating that this reduced accuracy reflects a consistent effect across trials at 400 samples rather than instability in the simulation or clustering procedure. The mechanism responsible for this length-dependent behavior remains unclear and requires further investigation. For the Logistic map (Figure 7c), clustering accuracy improves systematically with increasing time-series length, rising from 88.9% to 94.3%, while variability decreases markedly. Longer trajectories yield increasingly stable RT distributions, enhancing separability between parameter regimes. Similarly, the AR(2) process (Figure 7d) shows monotonic improvement, with accuracy exceeding 95% at the longest length. Unlike chaotic systems, variability remains low even for short sequences, reflecting the reproducible recurrence structure induced by linear stochastic dynamics.

#### 3.1.6. Statistical Comparison of RT and Baseline Methods

To assess differences among the three feature representations, clustering accuracies from the 50 independently generated trials were treated as the observational units for statistical comparison. Because the RT, RQA, and StatACF analyses used independently generated realizations, the resulting accuracy samples were treated as independent. Specifically, the analysis pipelines differ in random seeding and in the structure of the initial conditions sampled per trial; as a result, trial *i* does not refer to the same underlying realization across the three methods. Pairwise comparisons were therefore performed using the Wilcoxon rank-sum test (Mann–Whitney U test). A Kruskal–Wallis test was first used as an omnibus test to assess differences among the three methods, followed by pairwise comparisons of RT versus RQA, RT versus StatACF, and RQA versus StatACF. For each system-by-signal-length condition, Holm–Bonferroni correction was applied across these three pairwise comparisons. Independent-sample Cohen’s d was used to quantify effect size, and bootstrap 95% confidence intervals were calculated for the difference in mean accuracy.

The Kruskal–Wallis test indicated significant differences among the three methods at all tested system-by-signal-length conditions (all p < 0.001). Pairwise comparisons are summarized in Table 4, while the complete length-resolved results, including the RQA versus StatACF comparisons, are provided in Supplementary Data S1. Bootstrap 95% confidence intervals for each method’s individual accuracy estimate, at every system and signal length, are provided in Supplementary Data S2.

Compared with RQA, RT achieved significantly higher accuracy in 16 of the 20 system-by-signal-length conditions after Holm–Bonferroni correction. RT was significantly superior to RQA at every tested signal length for the Rössler system, Lorenz system, and Logistic map. For the AR(2) process, RT significantly outperformed RQA only at 200 samples; the difference at 400 samples was not significant, whereas RQA significantly outperformed RT at 600, 800, and 1000 samples.

Comparison with the StatACF baseline revealed a different, system-dependent pattern. StatACF significantly outperformed RT for the Rössler system at 400 samples and longer and for the Logistic map at every tested signal length. In contrast, RT significantly outperformed StatACF for the AR(2) process at every tested length. For the Lorenz system, RT significantly outperformed StatACF at four of the five signal lengths, whereas StatACF was significantly superior at 400 samples.

The magnitude of the observed differences was also reflected in the corresponding effect sizes and bootstrap confidence intervals. For example, at 400 samples for the Rössler system, RT exceeded RQA by 26.0 percentage points (95% CI: 23.6–28.2; d = 4.36), whereas StatACF exceeded RT by 9.3 percentage points (95% CI: −10.9 to −7.8; d = −2.31). The bootstrap confidence intervals for the accuracy differences excluded zero for the corresponding significant pairwise comparisons.

Overall, these results demonstrate that RT does not uniformly outperform the baseline representations. Instead, its relative clustering performance depends on both the underlying dynamical system and the available signal length. In particular, RT showed a consistent advantage over RQA for the Rössler system, Lorenz system, and Logistic map, whereas its performance relative to StatACF varied substantially across systems and signal lengths.

### 3.2. Results on Real Data

Human gait exhibits complex nonlinear dynamics, and dynamical systems approaches have been widely used to characterize gait variability, aging effects, and pathological locomotion [13,36,37,38].

#### 3.2.1. Gait Data and Participants

Gait data were collected in a controlled laboratory environment using a multi-camera motion-capture system with a fixed global coordinate frame. Each participant performed a continuous walking trial consisting of repeated out-and-back passes at a natural, self-selected pace under supervised conditions. The dataset comprised 19 individuals, including 10 older adults and 9 younger adults.

Three-dimensional trajectories of selected markers, including the waist, right ankle, and left ankle, were recorded in the laboratory’s fixed global coordinate frame. For each marker, raw spatial position coordinates (X, Y, Z, in meters)—rather than derived joint-angle kinematics—were obtained at each time frame and sampled uniformly at 200 Hz (sampling interval of 5 ms). Full trial durations ranged from approximately 33.7 to 41.7 s across the 19 participants; for consistency across participants, the first 6000 consecutive frames (30 s) of each recording were used for analysis. Individual gait cycles were not explicitly segmented or aligned. Each recording was analyzed as a single continuous trajectory over a fixed-duration window. Thus, the analysis used a fixed-duration signal rather than a fixed number of gait cycles. No filtering, detrending, spatial normalization, or temporal alignment was applied to the marker trajectories. The temporal ordering of the truncated sequence was retained in its original order. No missing marker values were present in the analyzed segments.

The older and younger groups differed significantly in age and height, whereas no statistically significant differences were observed in weight, BMI, or sex distribution (Table 5).

#### 3.2.2. Results Using RQA Features

We first evaluated whether conventional RQA descriptors can discriminate between younger and older participants based on gait dynamics. For each participant, six standard RQA measures were extracted from the RP of the gait time series: (a) determinism (DET), (b) average diagonal line length (L), (c) maximum diagonal line length ($L_{max}$), (d) laminarity (LAM), (e) entropy (ENTR), and (f) trapping time (TT). RQA measures were calculated separately for the waist, right ankle, and left ankle trajectories, with each marker treated as a single three-dimensional trajectory. The three resulting sets of six measures were concatenated into an 18-dimensional feature vector per participant.

Figure 8a shows the cluster assignments obtained from unsupervised k-means clustering (*k* = 2) in the RQA feature space; Figure 8b shows the same participants according to their known age groups, with circles indicating participants whose cluster assignment did not correspond to their reference age-group label. This solution correctly grouped 10 of the 19 participants, corresponding to an accuracy of 52.6%.

To assess the sensitivity of RQA-based clustering to *k*-means initialization, a separate analysis repeated the clustering procedure 100 times using different random initializations, with a standardized configuration (10 internal replicates) matching that used for the RT and StatACF comparisons. Across these 100 repeated runs, mean clustering accuracy was 55.26 ± 4.76% (range: 52.63–68.42%), with a mean adjusted Rand index of −0.02 ± 0.04, consistently near or slightly below zero across the repeated runs—indicating chance-level agreement with the true age-group labels rather than a reliably learnable cluster structure. Participant-level bootstrap resampling (10,000 iterations) provided a mean accuracy of 64.9% (95% CI: 52.6–89.5%), and a permutation test (10,000 label permutations) found no statistically significant association between the clustering and the age-group labels (*p* = 0.178) (Table 6).

Overall, conventional RQA features showed limited and initialization-sensitive agreement with the age-group labels in this small cohort. These results motivated the evaluation of the proposed recurrence-triangle (RT) features, described in Section 2, which provide a more localized geometric characterization of recurrence structures.

#### 3.2.3. Results Using the StatACF Method

We next evaluated the performance of a statistical and autocorrelation-based (StatACF) feature representation for distinguishing between younger and older participants based on gait dynamics. For each participant, statistical and autocorrelation-based features were extracted from the gait time series: the mean, standard deviation, skewness, kurtosis, and the first 10 autocorrelation coefficients were computed for each spatial coordinate (X, Y, Z) of the three selected gait markers, yielding 126 features per participant.

Figure 8c shows the cluster assignments obtained from unsupervised *k*-means clustering (*k* = 2) in the StatACF feature space; Figure 8d shows the same participants according to their known age groups, with circles indicating participants whose cluster assignment did not correspond to their reference age-group label. This solution correctly grouped 13 of the 19 participants, corresponding to an accuracy of 68.4%.

To assess the sensitivity of StatACF-based clustering to *k*-means initialization, the clustering procedure was repeated 100 times using different random initializations, with a standardized configuration of 10 internal replicates matching that used for the RQA and RT comparisons. Across these 100 repeated runs, mean clustering accuracy was 72.58 ± 3.84% (range: 52.63–73.68%), with a mean adjusted Rand index of 0.173 ± 0.051 and a mean normalized mutual information of 0.309 ± 0.078, indicating overall positive agreement with the reference age-group labels, although some variability across initializations was observed. Participant-level bootstrap resampling (10,000 iterations) provided a mean accuracy of 67.0% (95% CI: 52.6–89.5%), and a permutation test (10,000 label permutations) found no statistically significant association between the clustering and the age-group labels (*p* = 0.140) (Table 6).

Overall, the StatACF representation showed moderate and reasonably consistent agreement with the age-group labels in this small cohort, outperforming the RQA baseline in average clustering accuracy and showing lower variability across k-means initializations. However, the non-significant permutation result indicates that this clustering should not be interpreted as strong evidence of a reliable age-group structure on its own. These results motivated a direct comparison with the proposed RT-based framework, described in the following section, to determine whether a more localized geometric characterization of recurrence structure could achieve both higher and more stable clustering performance.

#### 3.2.4. Results Using the Proposed Method for Gait Data

We finally evaluated the proposed recurrence-triangle (RT) feature representation for distinguishing between younger and older participants based on gait dynamics. Unlike conventional RQA descriptors or StatACF features, the RT framework characterizes local geometric recurrence structures by analyzing the distribution of RTs extracted from RPs. RT features were computed independently from the waist (SACR), right ankle (RANK), and left ankle (LANK) trajectories, and the resulting feature vectors were concatenated to form a 9-dimensional feature representation for each participant. For each marker, we used *L* = 3, *RR* = 0.1, and *M* = 3, consistent with the parameter settings used for the Rössler system and the Logistic map. The three marker locations were analyzed independently to preserve location-specific recurrence information. The resulting feature vectors were then concatenated to form the combined representation.

Figure 8e shows the cluster assignments obtained from unsupervised k-means clustering (*k* = 2) in the RT feature space; Figure 8f shows the same participants according to their known age groups, with circles indicating participants whose cluster assignment did not correspond to their reference age-group label. This solution correctly grouped 14 of the 19 participants, corresponding to a clustering accuracy of 73.7%, the highest observed clustering accuracy among the three feature representations evaluated. To evaluate the robustness of the RT representation to *k*-means initialization, the clustering procedure was repeated 100 times using different random initializations, with the same standardized configuration (10 internal replicates) used for the RQA and StatACF analyses. Across all 100 repeated runs, clustering accuracy remained 73.68 ± 0.00%, with every run converging to an identical clustering solution (ARI = 0.180 ± 0.000; NMI = 0.169 ± 0.000; silhouette coefficient = 0.612 ± 0.000 in every run). These results indicate that the RT feature representation produced a stable, reproducible clustering solution that was entirely unaffected by *k*-means initialization. Participant-level bootstrap resampling (10,000 iterations) provided a mean clustering accuracy of 69.1% (95% CI:  52.6–94.7%). A permutation test based on 10,000 random permutations of the age-group labels yielded p = 0.073—numerically lower than the corresponding values for RQA (p = 0.178) and StatACF (p = 0.140), though this comparison is descriptive rather than a formal statistical test between methods—and did not reach the conventional 0.05 significance threshold. The robustness results for the RT representation across the 100 repeated *k*-means initializations are summarized in Table 6.

Overall, the RT representation achieved the highest observed clustering accuracy. It was also the only method that showed complete stability across *k*-means initializations, unlike RQA and StatACF. Pairwise McNemar tests found no significant difference between any pair of methods (all p≥0.375) (Table 7), likely because too few participants (n = 19) were classified differently to detect a difference. Even so, RT was more stable with respect to k-means initialization than RQA or StatACF in this dataset.

#### 3.2.5. Comparison Across Feature Representations

Table 6 summarizes the robustness of the RQA, StatACF, and RT feature representations across 100 repeated k-means initialization runs. RT achieved the highest mean clustering accuracy (73.68 ± 0.00%), followed by StatACF (72.58 ± 3.84%) and RQA (55.26 ± 4.76%). The RT accuracy remained unchanged across all repeated initializations, whereas RQA and StatACF showed variability across runs. RT also showed the highest mean silhouette coefficient (0.612 ± 0.000), while the mean ARI and NMI were 0.180 ± 0.000 and 0.169 ± 0.000, respectively. The corresponding bootstrap estimates and permutation results are also shown in Table 6.

Table 7 presents pairwise McNemar tests comparing the primary participant-level clustering assignments. None of the three pairwise comparisons reached statistical significance after Holm correction (all Holm-corrected, p≥0.375). Thus, although RT produced the highest observed clustering accuracy, the differences between the three feature representations were not statistically significant in this cohort.

#### 3.2.6. Recurrence Triangle Motif Distributions Across Age Groups

To further characterize the RT representation, we examined the distribution of individual RT motifs across the younger and older participant groups. Each RT motif represents a local recurrence configuration involving three consecutive time indices. Effect sizes were calculated using Cohen’s d, Hedges’ g, and Glass’s ∆ [39] to describe differences in motif occurrence between the two age groups. Positive effect sizes indicate higher motif occurrence in the older group, whereas negative values indicate higher occurrence in the younger group.

As shown in Figure 9a, for the left ankle trajectory, RT types 2–7 showed positive effect sizes, whereas type 1 showed a negative effect size. A similar distribution was observed for the right ankle trajectory (Figure 9b). For the waist trajectory (Figure 9c), RT type 8 showed a positive effect size, whereas RT types 1–7 showed predominantly negative effect sizes. These results indicate that the relative occurrence of RT motifs differed between age groups and varied according to marker location.

Figure 10 shows the reconstructed signal patterns associated with the eight possible RT configurations for *L* = 3. The reconstruction provides a visual representation of the temporal patterns associated with the different recurrence configurations.

## 4. Discussion

### 4.1. Comparison of RT with RQA and StatACF

Across the synthetic systems, RT generally provided stronger discrimination than RQA, although the magnitude and direction of the difference depended on the dynamical system and signal length. StatACF, however, outperformed RT under several conditions, including the Logistic map and parts of the Rössler and Lorenz ranges (Section 3.1). RT therefore does not universally outperform simpler alternatives. Instead, localized recurrence geometry appears to provide information that complements global recurrence statistics and conventional temporal descriptors.

RQA’s weaker performance may be related to its global representation of recurrence structure. RQA summarizes RPs using line-based statistics, which can be effective when systems differ in their overall temporal organization. When differences are expressed primarily through local recurrence configurations, these global statistics may be less sensitive to them. RT retains information about such local recurrence configurations, which may explain its stronger performance under some conditions.

As shown in Table 6, RT produced the highest observed gait clustering accuracy (73.7%), followed by StatACF (68.4%) and RQA (52.6%). However, this difference did not reach statistical significance: the RT permutation test gave *p* = 0.073, and none of the pairwise comparisons between the three representations were significant after Holm correction (Table 7).

RQA also showed a notable pattern: its relatively high silhouette coefficient was accompanied by very low agreement with the reference age labels. This illustrates that well-separated clusters in feature space do not necessarily correspond to the grouping of interest.

### 4.2. Interpretation of Gait RT Motifs

The motif redistribution reported in Section 3.2.6 is broadly consistent with prior reports of increased gait variability and reduced step-to-step coordination in older adults [41,42] and with reports of more stable locomotor patterns in younger adults [43]. The waist trajectory showed a different motif distribution from the ankle trajectories, indicating that the age-related redistribution of local recurrence structures was marker-dependent. However, the analysis used raw laboratory-frame trajectories. Walking path and turning behavior were not independently controlled, and walking speed was not measured. Therefore, these motif differences cannot be attributed specifically to age-related gait dynamics.

These interpretations remain exploratory. A single RT motif has no direct physiological meaning on its own. The reconstructed patterns in Figure 10 illustrate the recurrence configurations associated with each motif type but do not by themselves establish differences in gait stability or motor control. The present study did not measure stride-to-stride variability, step length, cadence, or walking speed, and these variables were not included as covariates. The two groups also differed significantly in height, which represents a potential confounding factor. Therefore, the motif distributions should be interpreted as statistical descriptors of recurrence structure rather than as direct markers of specific physiological mechanisms. Future work should combine RT features with gait-specific measures and larger cohorts to determine whether the observed motif differences persist after accounting for such factors.

We further assessed the statistical uncertainty of these motif-level effect sizes. We computed bootstrap 95% confidence intervals for Cohen’s d, Hedges’ g, and Glass’s ∆ for each of the 8 RT motifs at the three marker locations. Eighteen of the 24 confidence intervals excluded zero. This pattern was most consistent at the left ankle, where all 8 motifs had confidence intervals excluding zero, and least consistent at the waist, where 4 of 8 motif-level intervals included zero. A confidence interval excluding zero indicates that zero is outside the 95% bootstrap interval under the present analysis; it does not establish that the effect will replicate in an independent cohort. Accordingly, these results are interpreted as evidence of consistent motif-level differences within the present sample, rather than as evidence of clinically established or independently validated effects. Detailed bootstrap results are provided in Supplementary Data S3.

### 4.3. Clinical Relevance and Potential Applications

The observed gait clustering accuracy of 73.7% indicates that RT features can capture information associated with age-related differences in the present cohort. This performance should not be interpreted as sufficient for clinical diagnosis or decision-making: the difference between representations did not reach statistical significance, and the bootstrap confidence interval was wide. The present result is therefore preliminary rather than clinically validated. Nevertheless, RT’s ability to characterize localized recurrence structure suggests potential applications in clinically relevant gait analysis. Future studies could investigate RT-derived features as complementary descriptors for tasks such as fall-risk assessment, early detection of pathological gait patterns, or monitoring changes in gait dynamics over disease progression or rehabilitation. Such applications would require substantially larger, clinically characterized cohorts, longitudinal measurements, and direct comparison with established clinical gait measures. Whether the magnitude of RT feature differences is sufficiently large and reproducible to be clinically meaningful remains to be established. A statistically detectable difference is not automatically a clinically meaningful one; the present analysis found the RT advantage over StatACF and RQA did not reach statistical significance, indicating this question cannot yet be answered from the present data.

### 4.4. Limitations

Several factors limit the strength of the conclusions that can be drawn from the gait result. The cohort is small (n = 19), and the groups differed significantly in height, a potential confound not accounted for here. In addition, the analysis used raw, continuous marker trajectories without filtering, detrending, spatial normalization, temporal alignment, or gait-cycle segmentation. The recordings consisted of repeated out-and-back walking, which included turns. Consequently, the observed differences may partly reflect global translation, walking-path geometry, or turning behavior, rather than age-related gait dynamics alone.

The 100 repeated k-means initializations do not increase the effective sample size, since they re-analyze the same 19 participants. RT’s accuracy was identical across all 100 runs, indicating clustering stability rather than increased statistical power. The permutation test and pairwise comparisons did not reach conventional significance, and the bootstrap 95% confidence interval was wide (52.6–94.7%).

We therefore treat the gait result as a preliminary demonstration rather than validated evidence of age-related classification. The bootstrap estimate reflects resampling uncertainty within this cohort and does not test generalization to an independent sample. Runtime was not systematically measured in this study, so no claim of computational efficiency is made. Larger and more demographically balanced cohorts, gait-specific covariates, and external validation are needed before broader conclusions can be drawn.

## 5. Conclusions

This study introduced recurrence triangles (RTs) as a compact representation of local recurrence geometry for unsupervised time-series clustering. We compared RT with classical recurrence quantification analysis (RQA) and a statistical baseline (StatACF) across four synthetic systems and real gait data.

Across the synthetic systems, RT generally provided stronger discrimination than RQA, although the magnitude and direction of the difference depended on the system and signal length. RQA performed better than RT for AR(2) at longer signal lengths, while StatACF also outperformed RT under several conditions, including the Logistic map. No single representation was best in every case, suggesting that RT can provide complementary information about local recurrence structure that is not fully captured by global recurrence statistics and simple temporal descriptors.

In the gait data, RT achieved the highest observed clustering accuracy among the three representations (73.7%), but this difference was not statistically significant. We therefore treat this as a preliminary result rather than evidence of reliable age-group classification. RT motif distributions showed marker-dependent differences between the two age groups, but these differences should be treated as descriptive rather than physiological evidence without further validation.

This study has several limitations. The gait analysis was conducted on a small cohort, and the methodological comparison was limited to RQA and StatACF rather than a broader range of time-series clustering and representation-learning approaches. In addition, the RT features were not validated against clinical outcomes or biomechanical gait measures. Therefore, the present findings should be regarded as a proof-of-concept demonstrating the potential of RT representations for characterizing local recurrence structure, rather than as evidence of a validated clinical or general-purpose clustering tool.

Future work should evaluate RT across a wider range of noise levels, signal lengths, and preprocessing choices, and validate the gait findings using larger, independent, and more demographically balanced cohorts.

## Figures and Tables

**Figure 1:**
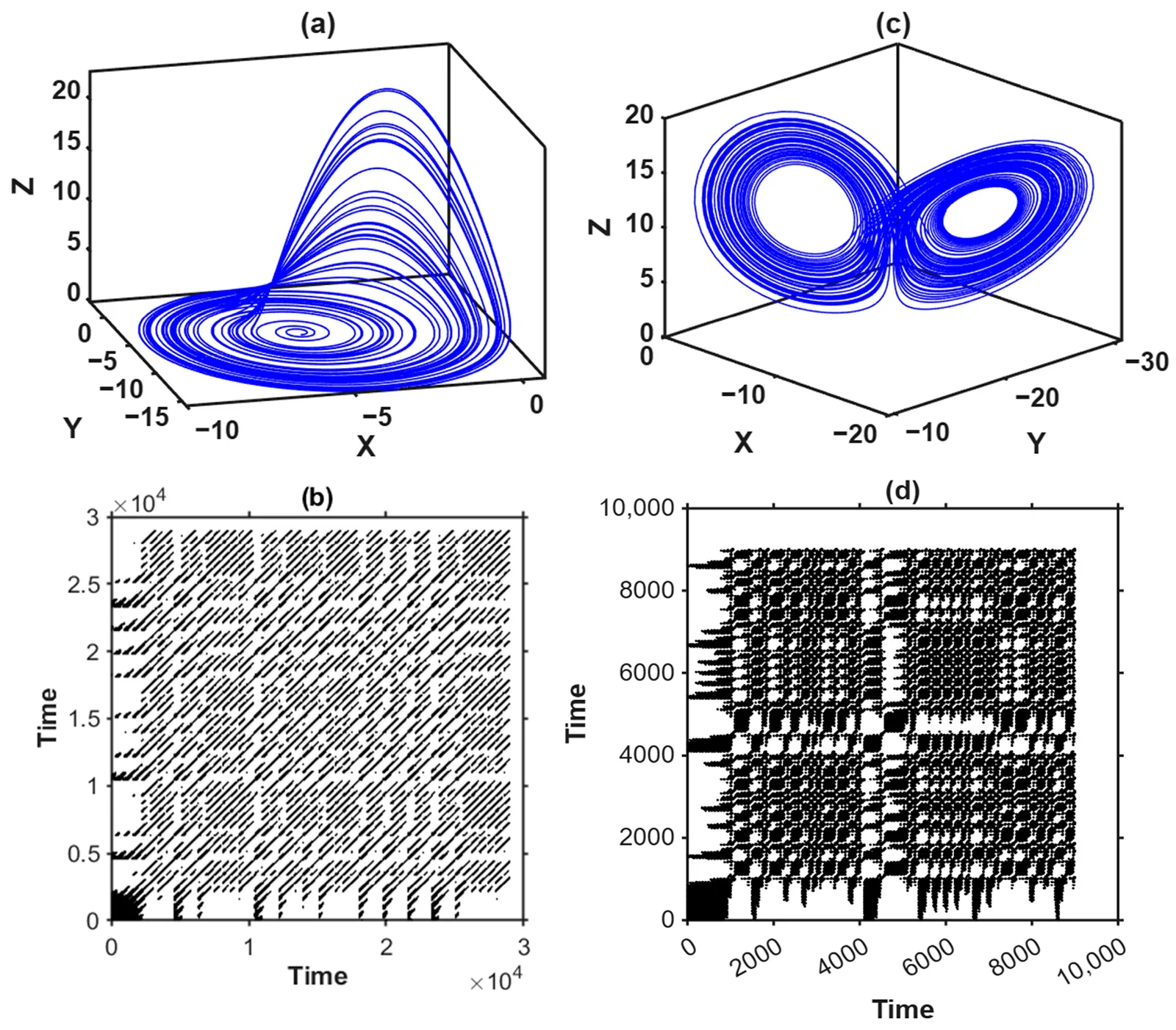
Phase-space trajectories and corresponding recurrence plots (RPs) for representative dynamical systems. Panels (**a**,**c**) show the attractors of the Rössler system and the Lorenz system, respectively. Panels (**b**,**d**) show their corresponding RPs.

**Figure 2:**
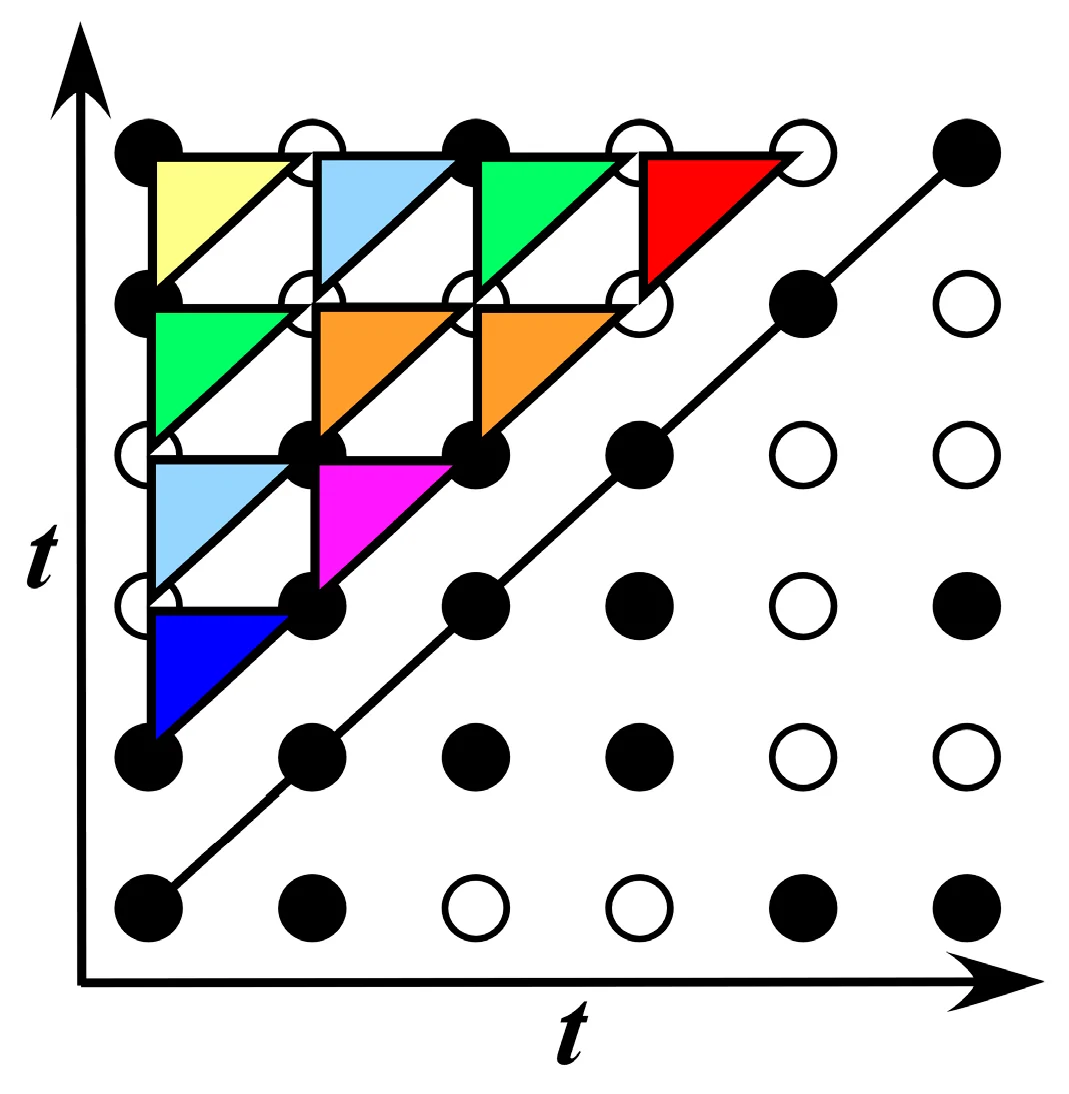
Illustration of recurrence triangle (RT) construction in a recurrence plot (RP). The upper triangular region of the RP is shown using RTs of fixed size *L* = 3 (colored regions). Each RT captures the local pairwise recurrence relationships.

**Figure 3:**
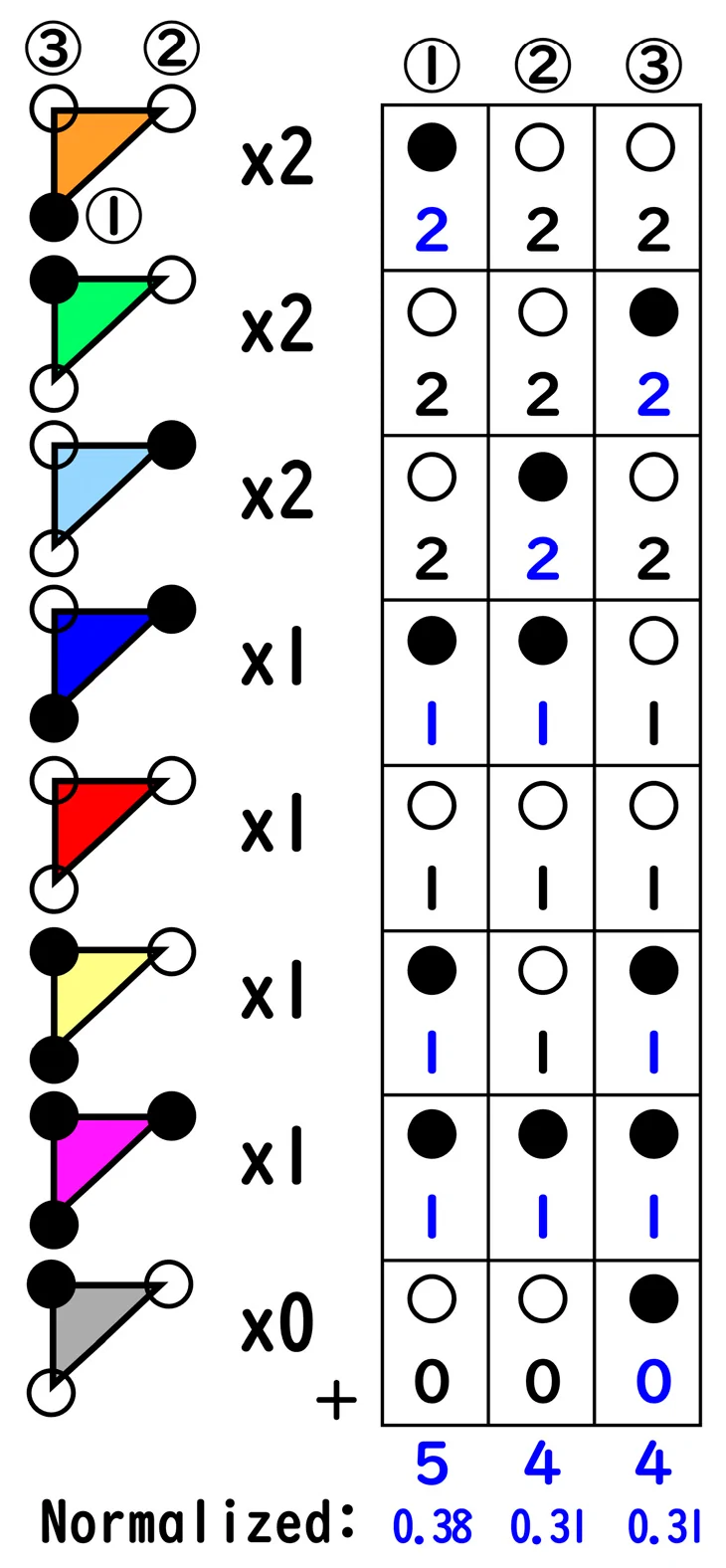
Example of recurrence triangle (RT) motif extraction and feature construction. Each RT configuration corresponds to a binary pattern based on pairwise recurrence relations. Identical patterns are grouped and counted, resulting in motif frequencies. These counts are aggregated to form a feature vector representing the distribution of RT motifs in the time series.

**Figure 4:**
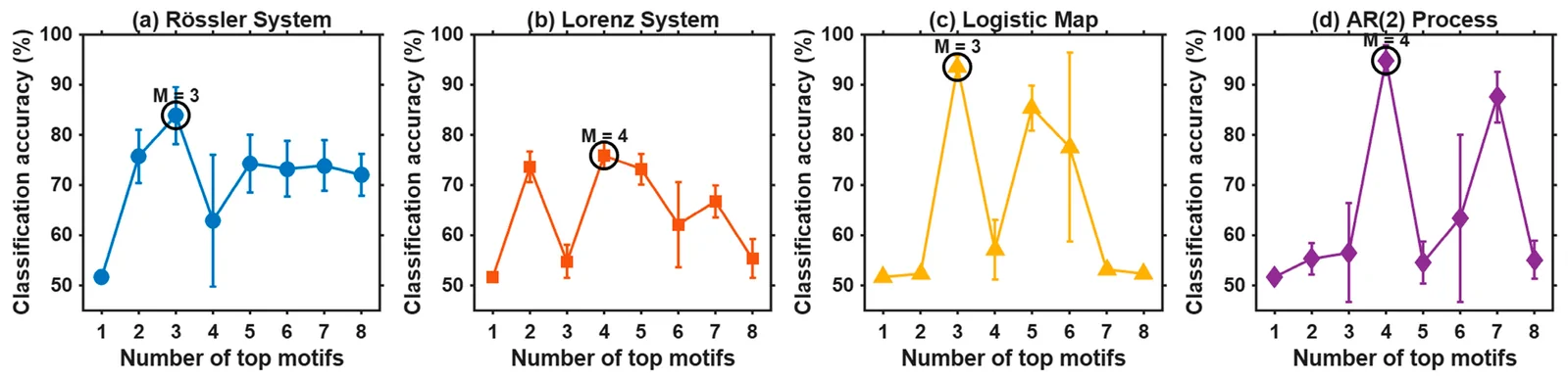
Sensitivity of RT-based clustering accuracy to the number of retained top motifs (*M*) for the four dynamical systems: (**a**) Rössler system, (**b**) Lorenz system, (**c**) Logistic map, and (**d**) AR(2) process. Triangle size was fixed at *L* = 3 and recurrence rate at *RR* = 0.10. Points indicate mean clustering accuracy across 50 independent trials, and error bars represent one standard deviation. Circled points indicate the selected *M* for each system. The selected *M* produced significantly higher accuracy than each alternative tested value after Holm correction (p<0.001); the selected values were also consistent across split-half stability analysis.

**Figure 5:**
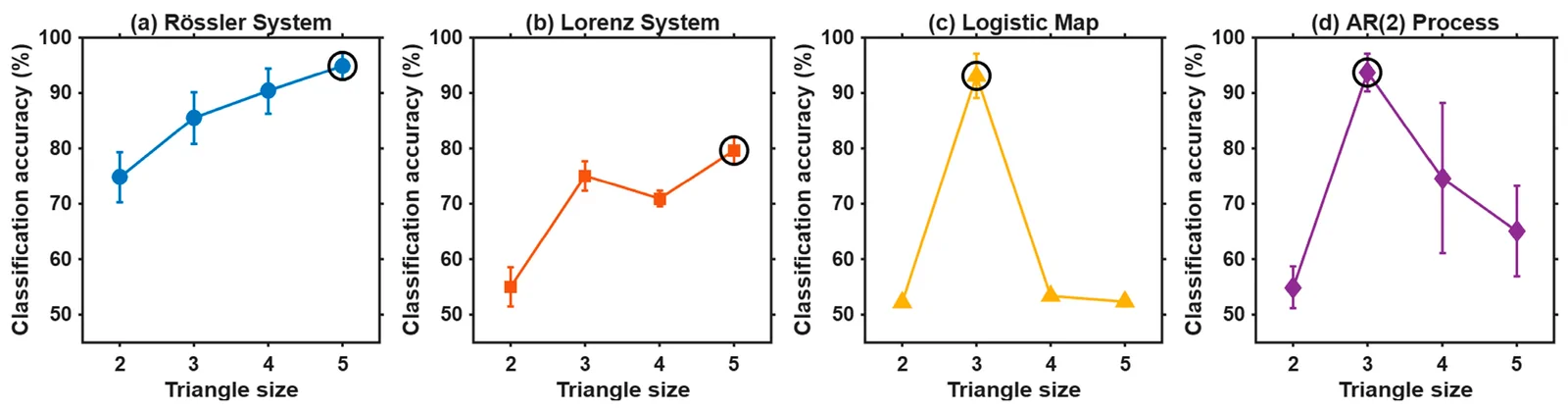
Sensitivity of RT-based clustering accuracy to triangle size (*L*) for (**a**) the Rössler system, (**b**) the Lorenz system, (**c**) the Logistic map, and (**d**) the AR(2) process. The recurrence rate was fixed at *RR* = 0.10, with *M* set to the value selected for each system in the section titled Selecting the top-*M* Parameter. Points show mean accuracy across 50 trials; error bars indicate one standard deviation. Circled points indicate the best-performing tested *L* for each system (*L* = 5 for Rössler/Lorenz; *L* = 3 for Logistic/AR(2)).

**Figure 6:**
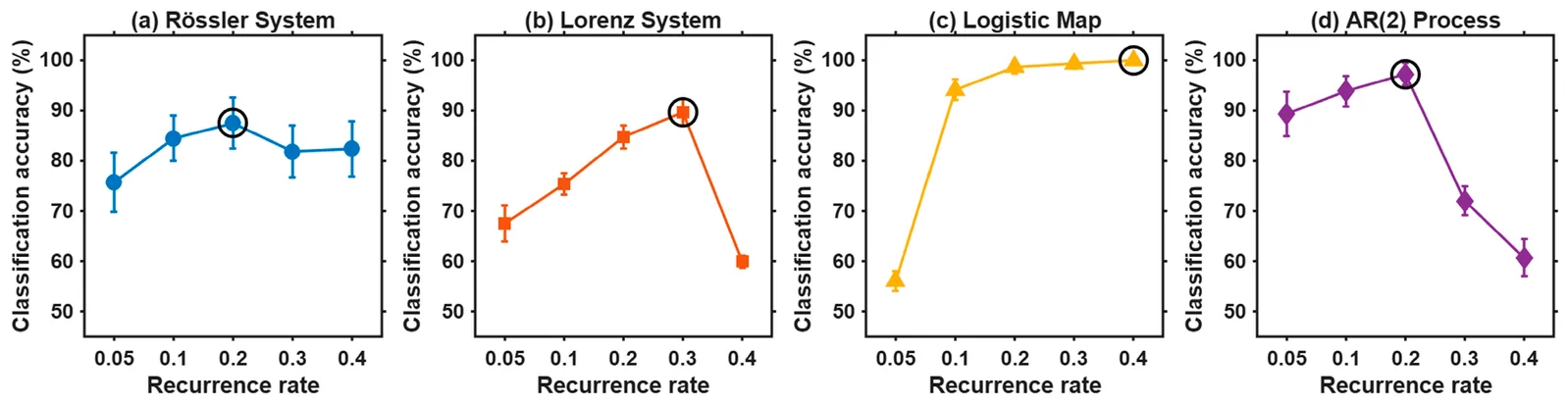
Sensitivity of RT-based clustering accuracy to recurrence rate (RR) for (**a**) Rössler system, (**b**) Lorenz system, (**c**) Logistic map, and (**d**) AR(2) process. Triangle size *L* = 3, with system-specific *M* selected from the *M*-sensitivity analysis. Points show mean accuracy over 50 independent trials; error bars indicate one standard deviation. Circled points indicate the highest-performing tested *RR* (*RR* = 0.20 for Rössler and AR(2), *RR* = 0.30 for Lorenz, and *RR* = 0.40 for Logistic).

**Figure 7:**
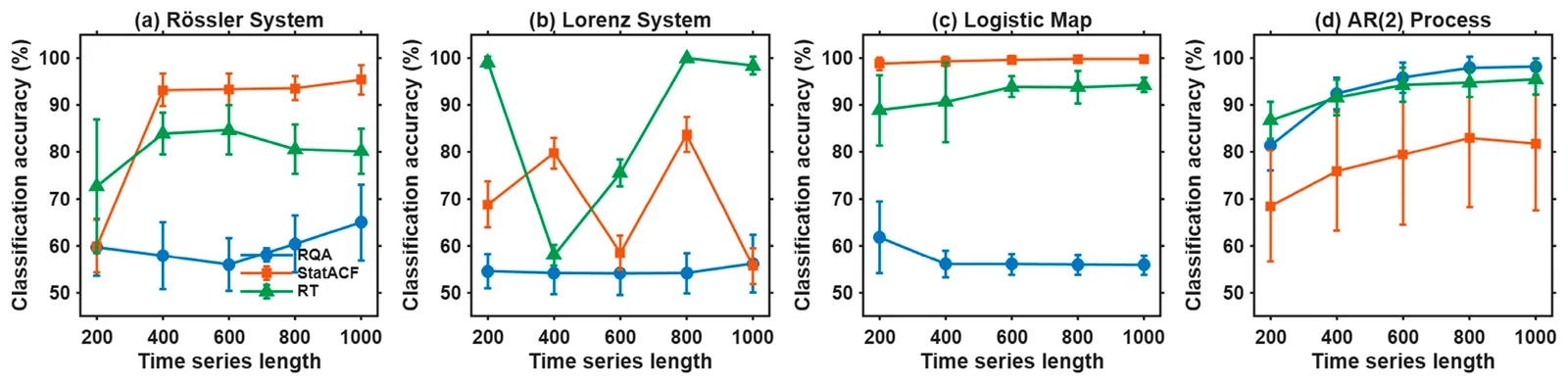
Comparison of clustering accuracy across time-series lengths for the RQA-, StatACF-, and RT-based methods applied to the (**a**) Rössler system, (**b**) Lorenz system, (**c**) Logistic map, and (**d**) AR(2) process. Each point denotes the mean accuracy obtained from 50 independently generated trials, with error bars representing the standard deviation across trials. The results demonstrate system- and length-dependent differences in the performance of the three feature-based approaches.

**Figure 8:**
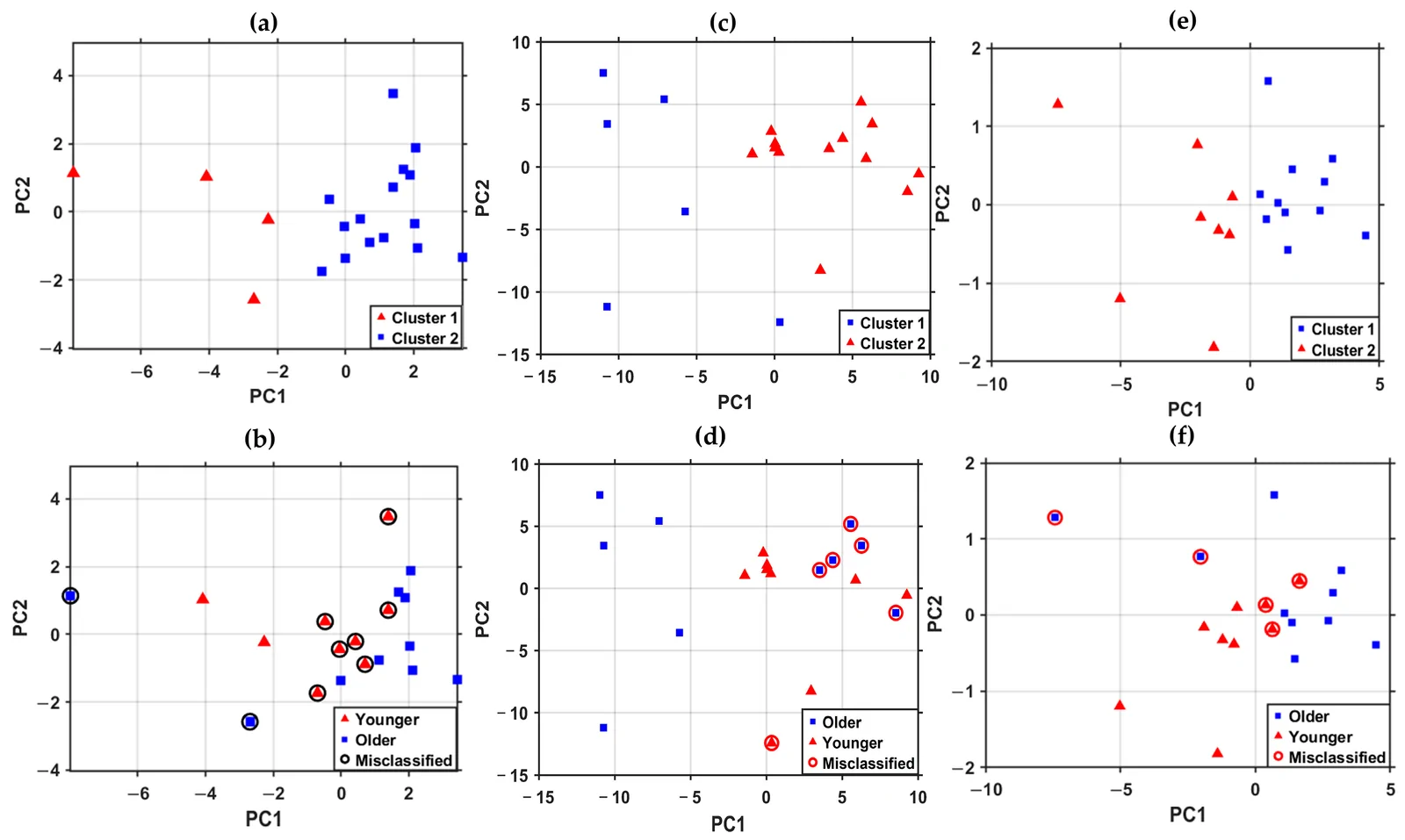
PCA visualization of unsupervised k-means clustering using RQA (**a**,**b**), StatACF (**c**,**d**), and RT (**e**,**f**) features. (**Top row**): resulting cluster assignments; (**bottom row**): the same participants colored by known age group, with circled points showing misclassifications. This figure reports a single clustering solution per method; robustness across repeated k-means initializations is reported separately in Table 6.

**Figure 9:**
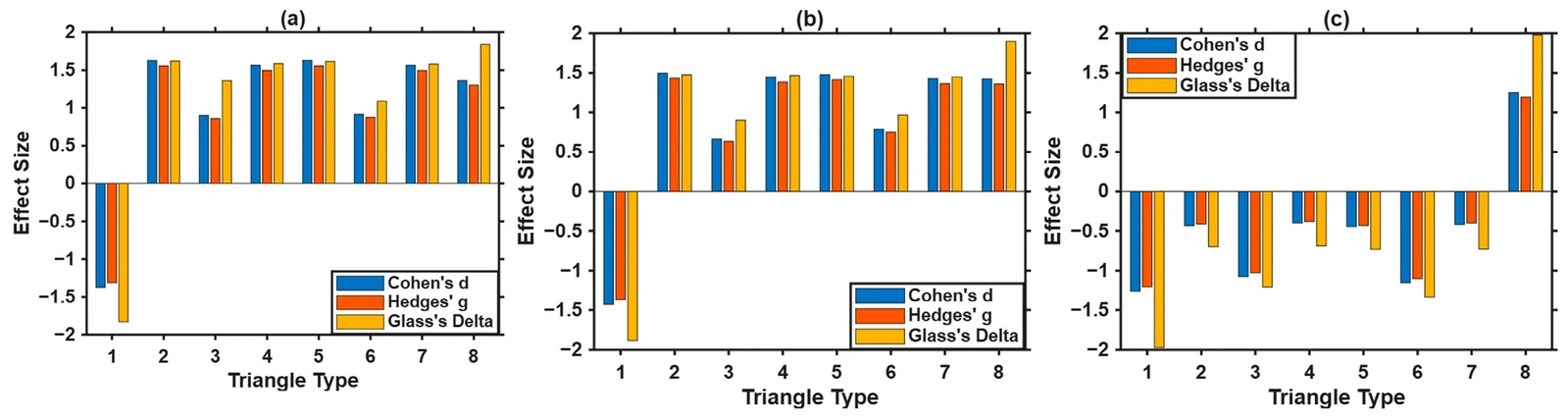
Effect size analysis of recurrence triangle (RT) motif distributions across age groups for three locations: (**a**) left ankle, (**b**) right ankle, and (**c**) waist. Effect sizes are computed using Cohen’s d, Hedges’ g, and Glass’s ∆ for each triangle type (1–8). Positive values indicate that the corresponding RT motif occurs more frequently in older participants, whereas negative values indicate higher occurrence in younger participants. At the ankles (**a**,**b**), triangle types 2–7 exhibit consistently positive effect sizes, indicating a higher prevalence in older participants, while type 1 shows negative values, indicating a higher occurrence in younger participants. In contrast, for the waist signal (**c**), triangle type 8 is more prevalent in older participants, whereas triangle types 1–7 are predominantly observed in younger participants. These results show descriptive, location-dependent differences in RT motif occurrence between the two age groups.

**Figure 10:**
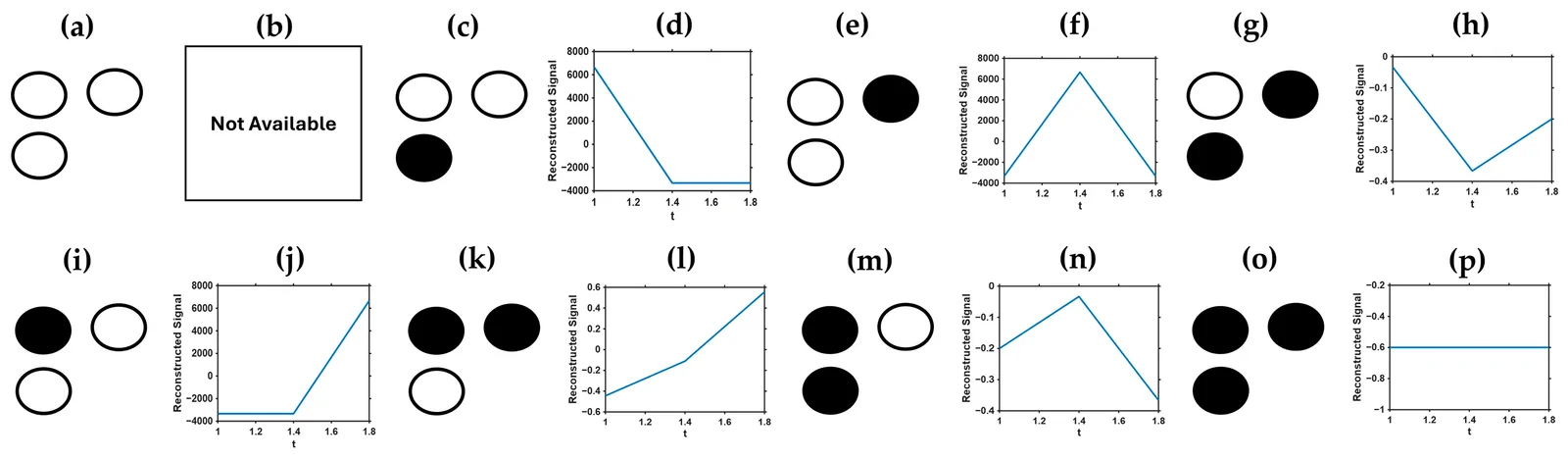
Reconstruction of time-series patterns corresponding to recurrence triangle (RT) motifs of size *L* = 3. Each motif represents a local recurrence configuration among three time indices. Panels (**a**,**c**,**e**,**g**,**i**,**k**,**m**,**o**) show the recurrence triangle patterns for RT motif Types 1–8, respectively, with filled circles denoting recurrence and open circles denoting non-recurrence; panels (**b**,**d**,**f**,**h**,**j**,**l**,**n**,**p**) show the corresponding reconstructed signals for each motif, in the same order. The reconstructed signals were obtained from the associated recurrence structure following the method of Hirata et al. [40].

**Table 1:** Clustering accuracy (%) of the RQA-based method across time series lengths.

System	200	400	600	800	1000
Rössler	59.7 ± 6	57.9 ± 7.1	56.0 ± 5.6	60.4 ± 6	65 ± 8.1
Lorenz	54.6 ± 3.6	54.2 ± 4.5	54.1 ± 4.6	54.2 ± 4.3	56.2 ± 6.1
Logistic	61.8 ± 7.7	56.1 ± 2.9	56.1 ± 2.2	56 ± 2.1	55.9 ± 2
AR(2)	81.4 ± 5.3	92.5 ± 3.4	95.9 ± 3.2	97.9 ± 2.4	98.2 ± 1.8

**Table 2:** Clustering accuracy (%) of the StatACF-based method across time series lengths.

System	200	400	600	800	1000
Rössler	59.9 ± 5.6	93.2 ± 3.5	93.4 ± 3.4	93.6 ± 2.6	95.4 ± 3.1
Lorenz	68.8 ± 4.9	79.8 ± 3.3	58.5 ± 3.7	83.7 ± 3.8	55.7 ± 3.8
Logistic	98.8 ± 1.3	99.3 ± 1.0	99.6 ± 0.8	99.8 ± 0.6	99.8 ± 0.5
AR(2)	68.5 ± 11.8	75.9 ± 12.6	79.4 ± 14.9	83.0 ± 14.7	81.8 ± 14.2

**Table 3:** Clustering accuracy (%) of the RT-based method across time series lengths.

System	200	400	600	800	1000
Rössler	72.7 ± 14.2	83.9 ± 4.5	84.7 ± 5.2	80.6 ± 5.3	80.1 ± 4.8
Lorenz	99.1 ± 1.1	58 ± 2.2	75.5 ± 2.9	100 ± 0.0	98.4 ± 1.9
Logistic	88.9 ± 7.5	90.6 ± 8.4	93.9 ± 2.2	93.8 ± 3.4	94.3 ± 1.5
AR(2)	86.7 ± 3.9	91.6 ± 3.8	94.3 ± 3.7	94.8 ± 3	95.5 ± 3.2

**Table 4:** Summary of pairwise statistical comparisons among RT, RQA, and StatACF across signal lengths (200–1000 samples). Comparisons were corrected for multiple testing within each system-by-signal-length condition using the Holm–Bonferroni procedure (three pairwise comparisons per condition).

System	Comparison	Outcome Across Lengths	∆Accuracy Range (pp)	Cohen’s *d* Range
Rössler	RT vs. RQA	RT significant 5/5	+13.0 to +28.8	1.19–5.34
Rössler	RT vs. StatACF	RT significant 1/5; StatACF significant 4/5	−15.2 to +12.8	−3.80–1.19
Lorenz	RT vs. RQA	RT significant 5/5	+3.9 to +45.8	1.09–17.75
Lorenz	RT vs. StatACF	RT significant 4/5; StatACF significant 1/5	−21.8 to +42.6	−7.86–14.26
Logistic	RT vs. RQA	RT significant 5/5	+27.0 to +38.4	3.55–22.06
Logistic	RT vs. StatACF	StatACF significant 5/5	−9.9 to −5.5	−4.98–−1.47
AR(2)	RT vs. RQA	RT significant 1/5; nonsignificant 1/5; RQA significant 3/5	−3.1 to +5.3	−1.13–1.14
AR(2)	RT vs. StatACF	RT significant 5/5	+11.7 to +18.2	1.11–2.07

**Table 5:** Demographic characteristics of the study cohort (n = 19), stratified by age group. Continuous variables (age, height, weight, and BMI) are reported as mean ± SD and compared between groups using Welch’s two-sample *t*-test; sex distribution was compared using Fisher’s exact test.

Variable	Older Adults (n = 10)	Younger Adults (n = 9)	*p*-Value
Age (years)	70.4 ± 4.5	29.9 ± 7.8	<0.0001
Height (cm)	158.8 ± 7.7	169.4 ± 9.7	0.019
Weight (kg)	60.8 ± 9.5	62.6 ± 5.4	0.623
BMI (kg/m^2^)	24.0 ± 2.5	21.9 ± 2.6	0.096
Sex (M/F)	5/5	5/4	1.000

**Table 6:** Robustness of the three feature representations across 100 repeated *k*-means initializations for the gait dataset (n = 19).

Method	Accuracy, Mean ± SD (%)	Range (%)	ARI, Mean ± SD	NMI, Mean ± SD	Silhouette, Mean ± SD	Bootstrap Mean [95% CI] (%)	Permutation p
RQA	55.26 ± 4.76	52.63–68.42	−0.021 ± 0.036	0.014 ± 0.029	0.578 ± 0.043	64.9 [52.6, 89.5]	0.178
StatACF	72.58 ± 3.84	52.63–73.68	0.173 ± 0.051	0.309 ± 0.078	0.379 ± 0.024	67.0 [52.6, 89.5]	0.140
RT	73.68 ± 0.00	73.68–73.68	0.180 ± 0.000	0.169 ± 0.000	0.612 ± 0.000	69.1 [52.6, 94.7]	0.073

**Table 7:** Pairwise McNemar tests comparing primary classifications between methods.

Comparison	Method A Only Correct	Method B Only Correct	Raw p	Holm-Corrected p
RT vs. RQA	4	0	0.125	0.375
RT vs. StatACF	6	5	1.000	1.000
RQA vs. StatACF	6	9	0.607	1.000

**Figure A1:**
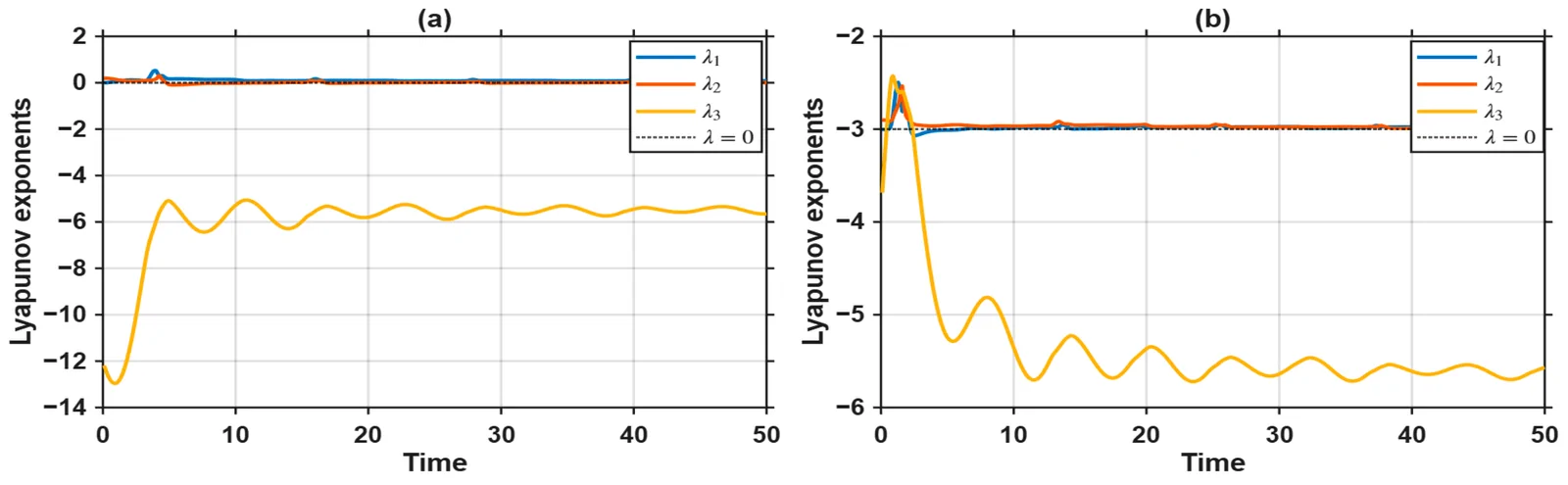
Convergence of the full Lyapunov exponent spectrum for representative Rössler parameter values. (**a**) Dataset 1 at b=0.405. (**b**) Dataset 2 at b=0.475. The solid curves represent the running estimates of $\lambda_{1},\;\lambda_{2},\;\lambda_{3}$, and the dotted horizontal line indicates $\lambda_{0}=0$.

**Figure A2:**
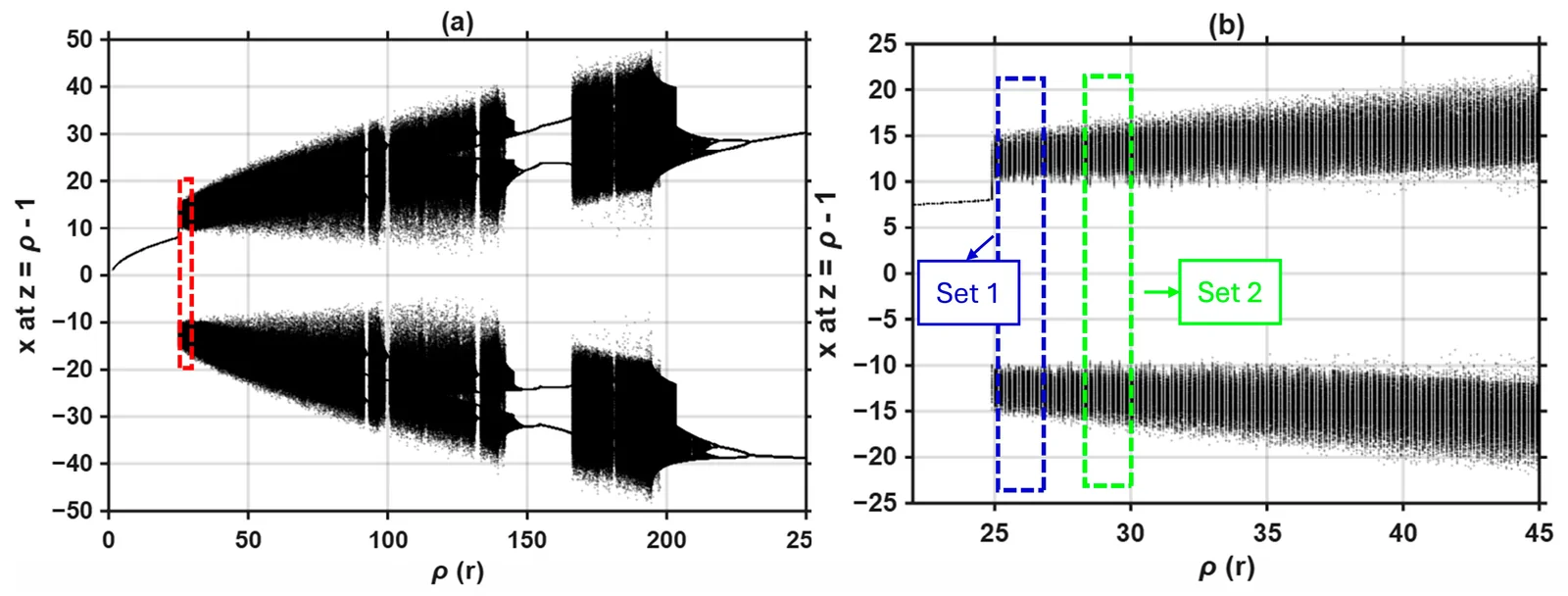
Bifurcation diagram of the Lorenz system (σ=10, $\beta =\;\frac{8}{3})$ based on the Poincare section at z= ρ−1. (**a**) Overview showing the transition to chaos. The red dashed box indicates the overall parameter range from which the two datasets were selected. (**b**) An enlarged view of the bifurcation range in which the first set and second set are clearly separated. First set range, ρ=25–27, shown in blue, and second set range, ρ=28–30, in green.

**Figure A3:**
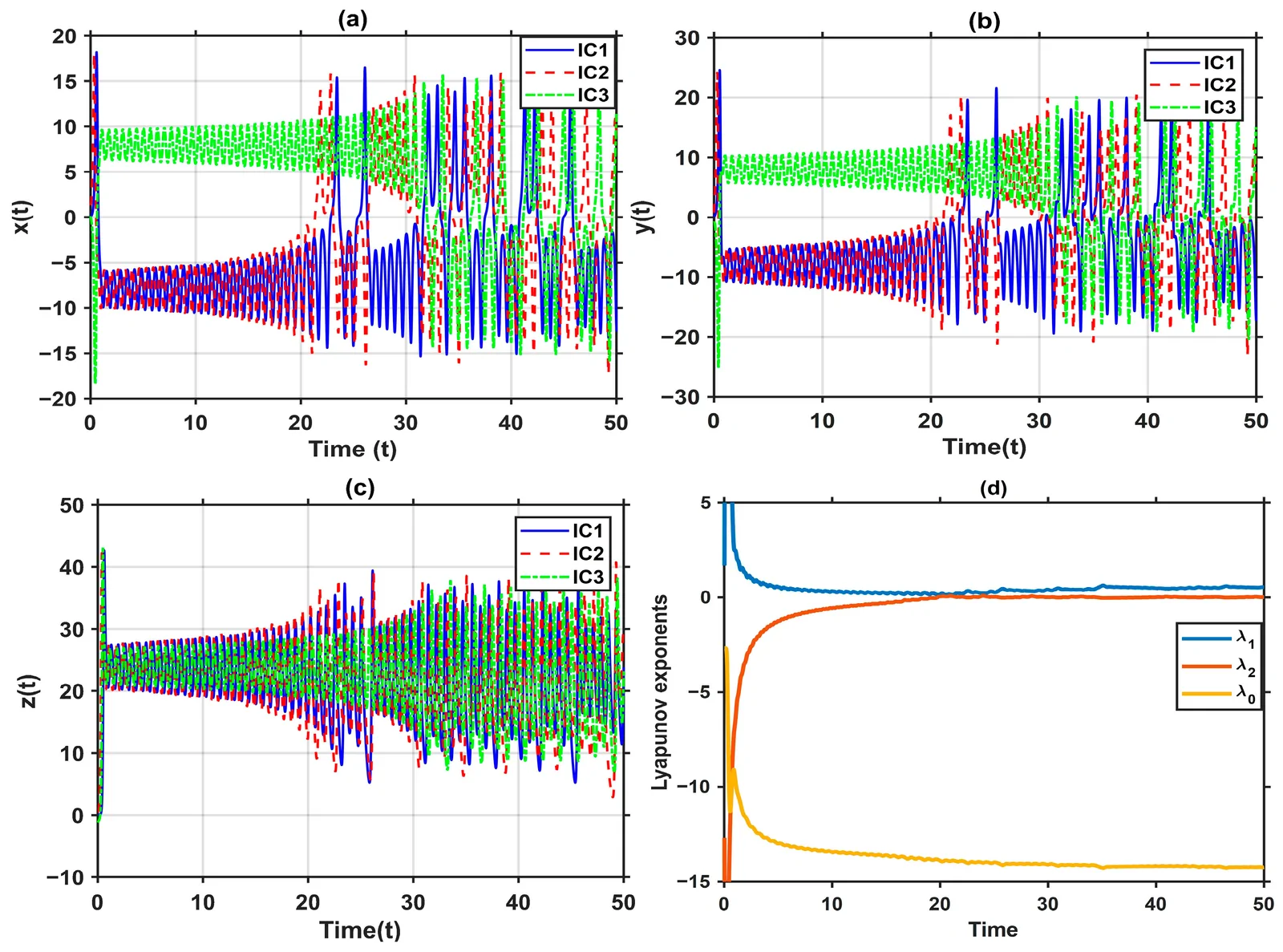
Demonstration of extreme sensitivity to initial conditions in the classical Lorenz system with fixed parameters σ=10, $\beta =\frac{8}{3}$, ρ=25, γ=0.1, and $\eta_{t}$ denoting Gaussian noise. (**a**) Time series of the x-component for three trajectories starting from nearby initial conditions. (**b**) Corresponding y-component time series. (**c**) Corresponding z-component time series. (**d**) Convergence of the corresponding Lyapunov exponent spectrum over time, showing the characteristic ordering $\lambda_{1}>0$, $\lambda_{2}\approx 0$, and $\lambda_{0}<0$. The reference initial condition is (0.1, 0.1, 0.1); the other two differ by small independent Gaussian perturbations (mean 0, standard deviation ≈ 0.1). All trajectories were integrated over a sufficient time interval after reaching the attractor (e.g., t ∈[0, 50]).

**Figure A4:**
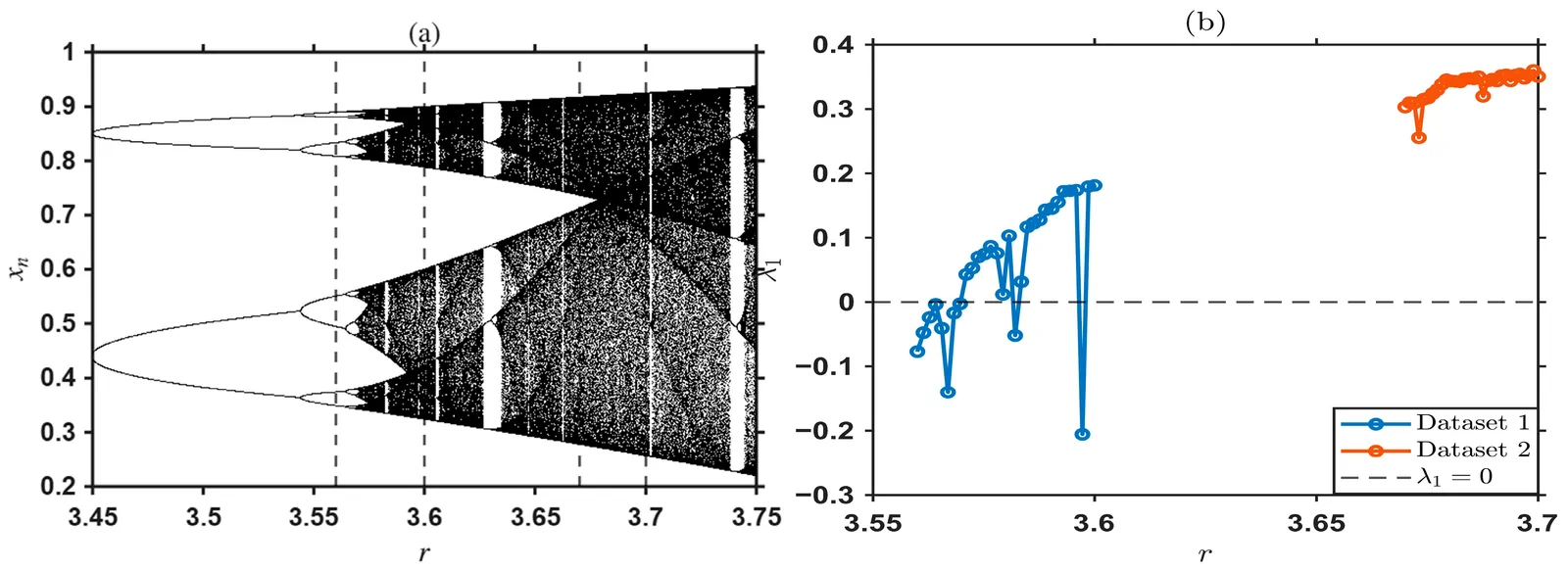
(**a**) Bifurcation diagram over 3.45≤r≤3.75, showing the transition from periodic to chaotic dynamics and the locations of the parameter intervals considered in this study. (**b**) Largest Lyapunov exponent $\lambda_{1}$ evaluated at the sampled parameter values for Dataset 1 and Dataset 2. The dashed horizontal line indicates $\lambda_{1}=0$; positive values indicate chaotic dynamics, whereas non-positive values indicate non-chaotic dynamics.

## Data Availability

Restrictions apply to the availability of these data. The data were obtained from the Research Institute on Human and Societal Augmentation (AIST) and are available from the corresponding author with the permission of the Research Institute on Human and Societal Augmentation (AIST). The data that support the findings of this study are available from the corresponding author upon reasonable request, subject to approval from the data owners.

## Supplementary Materials

The following supporting information can be downloaded at https://www.mdpi.com/article/10.3390/s26196137/s1. Supplementary Data S1: Complete statistical comparison results (Kruskal–Wallis, pairwise Mann–Whitney, Holm-Bonferroni correction, Cohen’s d, and bootstrap confidence intervals for accuracy differences) for all system-by-length conditions. Supplementary Data S2: Bootstrap 95% confidence intervals for individual method accuracy estimates (RT, RQA, StatACF) at every system and signal length. Supplementary Data S3: Bootstrap 95% confidence intervals for Cohen’s d, Hedges’ g, and Glass’s ∆ for the 8 RT motifs across the three marker locations.

## Appendix A

To validate the parameter choices used for the Rössler system, we performed complementary dynamical analyses confirming that the selected parameter ranges lie within distinct chaotic regimes.

### Appendix A.1. Bifurcation Analysis of the Rössler System

The Rössler bifurcation structure was previously investigated for the parameter configuration relevant to the present study. In particular, Lainscsek et al. [25] identified distinct dynamical ranges corresponding to b=0.37−0.44 and b=0.46−0.49, which were used to construct two dynamically distinct groups. The same parameter intervals are adopted in the present study.

### Appendix A.2. Lyapunov Exponent Analysis of the Rössler System

To quantitatively verify the chaotic character of the selected Rössler parameter ranges, the full Lyapunov spectrum ($\lambda_{1},\;\lambda_{2},\;\lambda_{3}$) was computed for all 30 sampled *b*-values in each dataset. The analysis was performed on the deterministic Rössler system without stochastic perturbations, thereby evaluating the underlying deterministic dynamical skeleton of the simulated system.

For Dataset 1, with b∈[0.37,0.44], the mean Lyapunov exponents were $\lambda_{1}$ = 0.0669±0.0112, $\lambda_{2}=0.0020\pm 0.0027$, and $\lambda_{3}=-5.4012\pm 0.0188$. For Dataset 2, with b∈[0.46,0.49], the corresponding values were $\lambda_{1}=0.0588\pm 0.0074$, $\lambda_{2}=0.0021\pm 0.0015$, and $\lambda_{3}=-5.3752\pm 0.0127$. All 30 parameter values in each dataset provided a positive largest Lyapunov exponent $\lambda_{1}>0$, providing numerical evidence of chaotic dynamics throughout both selected parameter intervals. In both datasets, the characteristic spectrum of a dissipative chaotic flow, $\lambda_{1}>0$, $\lambda_{2}\approx 0$, and $\lambda_{3}<0$, was consistently observed. A Mann–Whitney U test comparing $\lambda_{1}$ between the two parameter ranges yielded $p=6.91\times 10^{-4}$, with Cohen’s d = −0.852, indicating a statistically significant difference in the magnitude of the largest Lyapunov exponent between the two dynamically distinct parameter ranges (Figure A1).

## Appendix B

To validate the parameter choices used in the main experiments, we performed several complementary dynamical analyses to confirm that the selected parameter ranges lie within the chaotic regime of the classical Lorenz system. Specifically, we examined:

- The bifurcation structure as the control parameter ρ varies.
- The Lyapunov exponent spectrum, and
- The sensitivity of trajectories to initial conditions.

Together, these analyses confirm that the selected parameter intervals correspond to well-established chaotic dynamics of the Lorenz attractor.

### Appendix B.1. Bifurcation Analysis of the Lorenz System

We first examined the bifurcation structure of the Lorenz system by varying the control parameter ρ while fixing the classical parameters σ=10, $\beta =\;\frac{8}{3}.$ The system was numerically integrated over the interval $t\in \;\left[0,\;500\right]$, and transient dynamics were discarded. Figure A2 shows the resulting bifurcation diagram obtained using a Poincaré section at z= ρ−1. The diagram reveals several well-known transitions in the Lorenz system. A pitchfork bifurcation occurs at ρ=1, leading to two symmetric steady states. As ρ increases further, a Hopf bifurcation occurs near ρ≈24.74, after which the system transitions to sustained chaotic behavior.

The parameter ranges used in this study, ρ∈ [25, 27] and $\rho \;\in \;\left[28,\;30\right],$ lie well within the chaotic regime beyond the Hopf bifurcation, ensuring that both datasets correspond to chaotic attractors with distinct geometric structures.

### Appendix B.2. Lyapunov Exponent Analysis of the Lorenz System

To further confirm the presence of sustained chaos, we computed the Lyapunov exponent spectrum for representative parameter values within the selected intervals.

For the Lorenz system with σ=10 and $\beta =\;\frac{8}{3}$, the Lyapunov exponents exhibit the characteristic ordering $\lambda_{1}>0$, $\lambda_{2}\approx 0$, and $\lambda_{3}<0$. The positive largest Lyapunov exponent indicates exponential divergence of nearby trajectories, confirming the presence of deterministic chaos. Similar exponent structures were observed across the parameter range ρ∈[25, 30], indicating that the selected parameter intervals remain within the chaotic regime.

### Appendix B.3. Sensitivity to Initial Conditions of the Lorenz System

To examine sensitivity to initial conditions, we simulated trajectories starting from nearby initial states under identical parameter values. The Euclidean distance between trajectory pairs was monitored over time.

Figure A3 shows the time evolution of the system for three trajectories initialized with slightly perturbed initial conditions. The trajectories initially diverge exponentially, consistent with the positive Lyapunov exponent, and subsequently saturate due to the bounded nature of the attractor.

This behavior confirms that the system exhibits strong sensitivity to initial conditions while evolving on a single chaotic attractor. Consequently, the recurrence structures analyzed in the main text reflect intrinsic properties of the attractor rather than artifacts arising from multiple basins of attraction.

## Appendix C

To validate the parameter choices used for the Logistic map, we performed complementary dynamical analyses confirming that the selected parameter ranges lie within different dynamical regimes.

### Appendix C.1. Bifurcation Analysis of the Logistic Map

The dynamical behavior of the Logistic map was examined by varying the control parameter *r* over the range 3.45≤r≤3.75, while iterating the map after removal of an initial transient. The resulting bifurcation diagram is shown in Figure A4. The diagram shows the well-known transition from periodic to chaotic dynamics through a cascade of period-doubling bifurcations, together with periodic windows embedded within the chaotic regime.

The parameter intervals initially selected for the two datasets were r∈[3.56, 3.60] for Dataset 1 and r∈[3.67, 3.70] for Dataset 2. These intervals were selected based on the established bifurcation structure of the Logistic map and previous studies [33,34]. The bifurcation diagram shows that the two intervals occupy dynamically different regions of the parameter space, with Dataset 2 located in a more consistently chaotic region.

Because visual inspection of a bifurcation diagram alone does not provide a quantitative criterion for excluding periodic windows, the largest Lyapunov exponent was subsequently calculated for every sampled parameter value. The results are presented in Appendix C.2.

### Appendix C.2. Lyapunov Exponent Analysis of the Logistic Map

To quantitatively characterize the dynamical regimes associated with the selected Logistic map parameters, the largest Lyapunov exponent $\lambda_{1}$ was calculated independently for all 30 uniformly sampled *r* values in each dataset. For the Logistic map, a positive largest Lyapunov exponent indicates exponential sensitivity to initial conditions and is therefore used as a numerical indicator of chaotic dynamics.

For Dataset 1, with r∈[3.56, 3.60], the estimated largest Lyapunov exponent ranged from −0.2051 to 0.1830, with a mean of 0.0542±0.1004. In contrast, Dataset 2, with r∈[3.67, 3.70], exhibited consistently positive Lyapunov exponents. The estimated values ranged from 0.2723 to 0.3606, with a mean of 0.3374±0.0197, and all 30 sampled parameter values yielded $\lambda_{1}>0$. Thus, the numerical analysis confirms that Dataset 2 lies entirely within a chaotic regime.

These results demonstrate that the bifurcation diagram and Lyapunov-exponent analysis provide complementary information. While the bifurcation diagram identifies the overall dynamical structure, the Lyapunov exponent provides a quantitative criterion for assessing the dynamical character at each sampled parameter value.
